# MtNF-YC6 and MtNF-YC11 are involved in regulating the transcriptional program of arbuscular mycorrhizal symbiosis

**DOI:** 10.3389/fpls.2022.976280

**Published:** 2022-09-28

**Authors:** Chen Deng, Chun-Jui Li, Chen-Yun Hsieh, Li-Yu Daisy Liu, Yi-An Chen, Wei-Yi Lin

**Affiliations:** ^1^ Department of Horticulture and Landscape and Architecture, National Taiwan University, Taipei, Taiwan; ^2^ Department of Agronomy, National Taiwan University, Taipei, Taiwan

**Keywords:** arbuscular mycorrhizal fungus, symbiosis, nuclear factor Y, MtNF-YC6, MtNF-YC11

## Abstract

Arbuscular mycorrhizal fungi are obligate symbionts that transfer mineral nutrients to host plants through arbuscules, a fungal structure specialized for exchange for photosynthetic products. *MtNF-YC6* and *MtNF-YC11*, which encode the C subunits of nuclear factor Y (NF-Y) family in *Medicago truncatula* are induced specifically by arbuscular mycorrhizal symbiosis (AMS). A previous study showed that *MtNF-YC6* and *MtNF-YC11* are activated in cortical cells of mycorrhizal roots, but the gene functions were unknown. Herein, we identified both MtNF-YB17 and MtNF-YB12 as the interacting partners of MtNF-YC6 and MtNF-YC11 in yeast and plants. *MtNF-YB17* was highly induced by AMS and activated in cortical cells only in mycorrhizal roots but *MtNF-YB12* was not affected. The formation of B/C heterodimers led the protein complexes to transfer from the cytoplasm to the nucleus. Silencing *MtNF-YC6* and *C11* by RNA interference (RNAi) resulted in decreased colonization efficiency and arbuscule richness. Coincidently, genes associated with arbuscule development and degeneration in RNAi roots were also downregulated. *In silico* analysis showed CCAAT-binding motifs in the promoter regions of downregulated genes, further supporting the involvement of NF-Y complexes in transcriptional regulation of symbiosis. Taken together, this study identifies MtNF-YC6- or MtNF-YC11-containing protein complexes as novel transcriptional regulators of symbiotic program and provides a list of potential downstream target genes. These data will help to further dissect the AMS regulatory network.

## Introduction

Arbuscular mycorrhizal fungi (AMF), the soil-borne fungi, belong to Glomeromycota, and can form symbiotic relationships with more than 80% of land plant species (Smith and Read, 2008). These fungi are obligate symbionts that acquire mineral nutrients through extraradical hyphal network and transfer them to host plants through the specialized fungal structure and the interface (the arbuscule and peri-arbuscular space, respectively) in the inner cortical cells in exchange for photosynthetic products from host plants (Roth and Paszkowski, 2017).

After perceiving the fungal signals, a series of calcium oscillations is induced. A nuclear-localized calcium calmodulin-dependent protein kinase (CCaMK, known as DMI3 in *Medicago truncatula*) acts as decoder to decipher the calcium oscillation and activate symbiotic responses and initiate the formation of prepenetration apparatus (Takeda et al., 2012; Miller et al., 2013). CCaMK phosphorylates and interacts with CYCLOPS (known as IPD3 in *M. truncatula*) to promote symbiosis (Yano et al., 2008; Horváth et al., 2011). Several GRAS domain transcriptional regulators act downstream of CCaMK to regulate symbiotic processes, such as NSP1 and NSP2 (Delaux et al., 2013a; Hofferek et al., 2014) and RAM1 (Park et al., 2015). Genes participated in arbuscule development have been identified, including *Vapyrin* (Pumplin et al., 2010; Murray et al., 2011), *RAM2* (Wang et al., 2012; Gobbato et al., 2013), *STR and STR2* (Zhang et al., 2010), but the expression pattern of these genes are varied, suggesting that the complex regulation programs are required to ensure gene expression precisely. Although arbuscules function as a platform for nutrient exchange, the life span of this fungal structure is relatively short (Alexander et al., 1988; Alexander et al., 1989). Thus, arbuscule degeneration and new arbuscule development occur simultaneously in mycorrhizal roots to maintain an active symbiotic relationship. Currently, the knowledge about the underlying mechanism of controlling arbuscule degeneration is limited.

*Medicago truncatula* PT4, an AM symbiosis (AMS)-responsive phosphate transporter, is localized in a specialized cortical cell membrane that encircles an arbuscule to take up fungal phosphate. Loss of MtPT4 function did not affect arbuscule development but triggered premature arbuscule degeneration leading to less phosphate accumulation (Harrison et al., 2002; Javot et al., 2007). During the degeneration process, peroxisomes accumulate around collapsing arbuscules either to assist lipid metabolism or sequester reactive oxygen species produced at this phase (Pumplin and Harrison, 2009). MtMYB1 is the first transcriptional regulator identified involved in controlling premature arbuscule degeneration. Reducing *MtMYB1* expression in the *mtpt4* mutant restored the premature arbuscule degeneration phenotype. This transcription factor coupled with NSP1 and DELLA, participated in the transcriptional regulation of hydrolase genes associated with arbuscule degeneration (Floss et al., 2017). However, the mechanism of initiating arbuscule degeneration and the transcriptional regulation upstream to MtMyb1-mediated transcriptional program remain unclear.

The symbiosis is believed to have arisen more than 400 million years ago (Remy et al., 1994). The set of essential genes required for the formation of AMS - a symbiotic toolkit - is highly conserved in many host species. These genes usually respond specifically to AMS and are thought to be involved in AMS formation and maintenance (Delaux et al., 2013b). Through comparative phylogenomic analyses, more than 100 candidate genes that are evolutionarily conserved in host species were identified, including well-characterized AMS-responsive genes and many uncharacterized genes (Delaux et al., 2014; Favre et al., 2014; Bravo et al., 2016). Among the AMS-conserved genes, 13% are involved in transcriptional regulation (Bravo et al., 2016), and *RAM1* and *CYCLOPS* were the most well-characterized transcriptional factors involved in controlling arbuscule development. (Park et al., 2015; Floss et al., 2016; Pimprikar et al., 2016). One of the CCAAT-binding factors (CBFs) was also identified as a AMS-conserved genes and highly induced by symbiosis (Delaux et al., 2014; Favre et al., 2014; Bravo et al., 2016), but the exact role remains unclear.

CCAAT-binding factors, also named Nuclear Factor Ys (NF-Ys) or Heme Activator Proteins (HAPs), are present in all eukaryotic species. These transcriptional regulators are composed of three subunits, NF-YA, NF-YB and NF-YC. In the heterotrimeric complex, NF-YA is responsible for specific recognition of the CCAAT-binding motif in the promoter regions. NF-YB and NF-YC contain histone fold motifs, form heterodimers and interact with NF-YA to bind to the core DNA sequence (Romier et al., 2003; Petroni et al., 2012). In plants, NF-Ys are encoded by multigenic families leading to numerous kinds of heterotrimeric combinations participating in various kinds of physiological processes and rhizobium-legume symbiosis (Petroni et al., 2012; Laloum et al., 2013; Ripodas et al., 2014; Baudin et al., 2015; Zhao et al., 2016), but very few reports have demonstrated the roles of NF-Ys in AMS. Soybean *GmNF-YA1a* and *1b* were downregulated by NARK-mediated autoregulation to further reduce AMF infection events, identified as positive regulators of AMS (Schaarschmidt et al., 2013). MtNF-YC6 and MtNF-YC11 (previously designated MtCbf1 and MtCbf2) have been identified as AMS-conserved transcriptional factors (Delaux et al., 2014; Favre et al., 2014; Bravo et al., 2016). Cell-specific transcriptomic analysis and promoter analysis revealed that both genes were first induced by the contact between fungi and host plants, and the expression was also detected in arbuscule containing-cortical cells and adjacent cells when fungal hyphae extended to the cortex (Hogekamp et al., 2011). These results imply that both genes might participate in controlling several symbiotic processes. However, their interacting partners and their roles in the AMS regulatory network await for further characterization.

In this study, we identified MtNF-YB12 and B17 as the interacting partners of MtNF-YC6/C11 and showed the activation of *MtNF-YB17* by AMS in the cortical cells of infection units. When knocking down *MtNF-YC6* and *MtNF-YC11* in mycorrhizal roots by RNA interference (RNAi), we observed a decrease in colonization efficiency and the downregulation of genes involved in the control of arbuscule development and degeneration. Furthermore, *in silico* analysis revealed the potential of these downregulated genes as the downstream targets of NF-Y complexes. Some well-characterized AMS-responsive genes also obtained more than one CCAAT-binding motif and were downregulated in RNAi roots, implying that NF-Y complexes might be involved in the control of different symbiotic processes. Our results provide new insights into the transcription program of arbuscule development.

## Materials and methods

### Plant growth conditions

*Medicago truncatula* ssp. *truncatula* ecotype Jemalong (A17) and *Nicotiana benthamiana* were used in these studies. The plants were grown in a growth chamber with 16-h light (25°C) and 8-h dark (22°C). *Medicago* seedlings were grown in cones filled with sterilized river sands and fertilized twice a week with a modified one-half strength of Hoagland’s solution containing 20 μM potassium phosphate. For AMF treatment, 1 g of *Claroideoglomus etunicatum* or *Rhizophagus irregularis* inoculants (containing around 100 spores) were added before transplanting. Plants were harvested 6 weeks later for further research. *N. benthamiana* were grown in pots filled with a mixture of peat moss and vermiculite in a 9:1 ratio and fertilized with full nutrient solution once a week. At 4 weeks after transplanting, leaves were used for agroinfiltration.

### *Medicago truncatula* root transformation

*M. truncatula* root transformation was conducted as described by Boisson-Dernier et al. (2001) with minor modifications. Seeds of *M. truncatula* were surface sterilized and kept in the dark at 4°C overnight. Then the seeds were transferred to germinate in a 30°C incubator for 15 h. The root tips of *Medicago* seedling were cut and co-cultivated with *Agrobacterium rhizogenes* strain Arqua1 harboring a binary vector. After cocultivation, plants were transferred to cones filled with sterilized river sands and 1 g of AMF inoculants and were grown for another 6 weeks before harvesting.

### Construction for knocking down the expression of MtNF-YC6/C11 and MtNF-YB17

For generating the *MtNF-YC6/C11* and *MtNF-YB17* RNAi constructs, around 300 bp of specific sequences in the coding regions (**Supplementary Figure 1**) were amplified using primer pairs listed in **Supplementary Table 1** and cloned into pDONR221 by Gateway BP reaction (Thermo Fisher Scientific, USA). These vectors were recombined with the pK7GWIWG(II)-RedRoot destination vector (S. Ivanov, unpublished) through Multisite Gateway LR reaction (Thermo Fisher Scientific) to generate RNAi transformation vectors under the control of the CaMV 35S promoter.

### Construction for transient gene expression in *Nicotiana. benthamiana* leaves

Genes of interest were amplified using primer pairs listed in **Supplementary Table 1**. For detecting the subcellular localization of MtNF-YCs, MtNF-YBs and MtNF-YAs, the coding sequences were recombined with pDONR221 in the Gateway BP reaction. To express N-terminal tagged fluorescence fusion proteins, pK7WGC2, pK7WGF2 and pK7WGY2 were used to generate CFP-, GFP-, and YFP-fused proteins through Gateway LR reaction, respectively (Karimi et al., 2002).

For bimolecular fluorescence complementation (BiFC) analysis, the coding sequence of *MtNF-YC*s and *MtNF-YB*s were recombined with pUBN-nYFP, pUBC-nYFP and pUBC-cYFP (Grefen et al., 2010) to generate N-terminal YFP and C-terminal YFP tagging at either N or C terminus of target proteins.

### *Agrobacterium tumefaciens*-mediated infiltration

*Agrobacterium*-mediated infiltration was performed as described (Liu et al., 2012). Briefly, the culture of *A. tumefaciens* EHA105 strain containing a binary vector was prepared in LB media incubating at 30°C overnight. The culture was resuspended in the infiltration medium (10 mM MgCl_2_ and 10 mM MES) and diluted to an OD_600_ of 1.0. The cell suspension was kept in the dark for 2-3 h at room temperature. A mixture of cell suspension containing genes of interests was infiltrated into the leaves of *N. benthamiana*. Samples were collected 3-4 days after infiltration.

### WGA staining and analysis of colonization efficiency

*Medicago* roots were cut into 1-cm fragments and stained with WGA-Alexa fluor 488 (Thermo Fisher Scientific) to visualize fungal structures (Park et al., 2015). The root fragments were selected randomly and examined microscopically using an Olympus SZX16 stereomicroscope (Olympus, Japan). The grid method evaluated the percentage of infected roots containing fungal structures (F%; Mcgonigle et al., 1990), the intensity of mycorrhization in the root systems (M%) and in mycorrhizal root fragments (m%), and the arbuscule abudnace in the root systems (A%) and in root fragments containing arbusucles (Trouvelot et al., 1986). The arbuscule distribution was analyzed as described (Breuillin-Sessoms et al., 2015). The arbuscules were classified based on their length and the abundance of arbuscules in difference classes was calculated as percentage.

### Yeast two-hybrid assay and plasmid construction

The yeast two-hybrid assay was conducted according to the manufacturer’s instructions (Clontech, USA). For testing the interaction between NF-YBs and NF-YCs, the coding sequences of *MtNF-YCs* and *MtNF-YBs* were cloned into pGBKT7-DEST and pGADT7-DEST, respectively. For testing the interaction between NF-YAs and NF-YCs, *MtNF-YC6*/*C11* and *MtNF-YA*s were cloned to pGADT7-DEST and pGBKT7-DEST, respectively. Primer pairs used for amplifying genes of interest are listed in **Supplementary Table 1**. Yeast cells cotransformed with pGADT7-T and pGBKT7-53 were positive controls while cells cotransformed with pGADT7-T and pGBKY7-Lam were negative controls. The specificity of protein-protein interactions was confirmed by the growth of yeast cells on dropout media lacking leucine, tryptophan, and histidine and supplemented 3-amino-1,2,4-triazole (3AT).

### Construction for promoter analysis and GUS-staining

For analyzing the activity of the *MtNF-YB17* promoter, a 1.25-kbp fragment upstream of the start codon was amplified using primer pairs listed in **Supplementary Table 1** and was cloned into pBGWFS-RedRoot which was kindly provided by Dr. Shu-Yi Yang (National Taiwan University, Taiwan).

*Medicago* roots were transformed with *MtNF-YB17pro:GUS* and composite roots were collected at 6 weeks after transplanting for staining. The roots were fixed in ice-cold 90% acetone for 30 min and washed three times with phosphate buffered saline (PBS). Then, the roots were incubated in GUS staining buffer (5 μM EDTA, 0.5 mM potassium ferricyanide. 0.5 mM potassium ferrocyanide and 0.5 mg ml^-1^ 5-bromo-4-chloro-3-indolyl-β-D-glucuronide cyclohexylammonium salt) at 37°C for 6 h and washed with PBS to stop the reaction. The stained roots were observed under an Olympus SZX16 stereomicroscope and a Zeiss Axio Imager M2 light microscope (Zeiss Microscopy, Germany).

### Fluorescence microscopy

Fluorescence images were taken by confocal microscopy using a Leica TCS SP5 II (Leica Microsystems, Germany) with objectives HCX PL FLUOTAR 10X/0.30 DRY, HCX PL APO CS 20X/0.7 dry and HCX PL APO lambda blue 63X/1.40 OIL. The excitation and emission wavelengths for CFP were 458 nm and 465-510 nm; 475 nm and 500-520 nm for GFP; 514 nm and 520-550 nm for YFP.

### RNA extraction and gene expression analysis

Total RNA was isolated using TRIzol^®^ (Thermo Fisher Scientific) according to the manufacturer’s instruction and treated with RNase-free TURBO DNase I (Thermo Fisher Scientific) to remove genomic DNA. First strand cDNAs were synthesized from 500 ng RNA using Moloney murine leukemia virus reverse transcriptase (Thermo Fisher Scientific) with oligo dT primer. Quantitative RT-PCR (qRT-PCR) was performed using iQ™ SYBR^®^ Green Supermix (Bio-Rad, USA) on a CFX Connect Real-Time PCR Detection System (Bio-Rad). Relative expression levels (2^-ΔCt^) were normalized to the expression of a reference gene, *MtEF1-α*. The primer pairs used for qRT-PCR are listed in **Supplementary Table 2**.

### *In silico* analysis of CCAAT-binding motifs

The promoter sequences (2 kbp upstream of the start codon) of 91 differentially expressed genes in *mtpt4* mutants (Floss et al., 2017) and AMS marker genes were retrieved from *Medicago truncatula* genome database (Tang et al., 2014). The R program (version 4.1.0) was used to scan and identify CCAAT-binding motifs in the promoter regions.

## Results

### Identifying the NF-Y subunits that interact with MtNF-YC6 and MtNF-YC11

**Figure 1:**
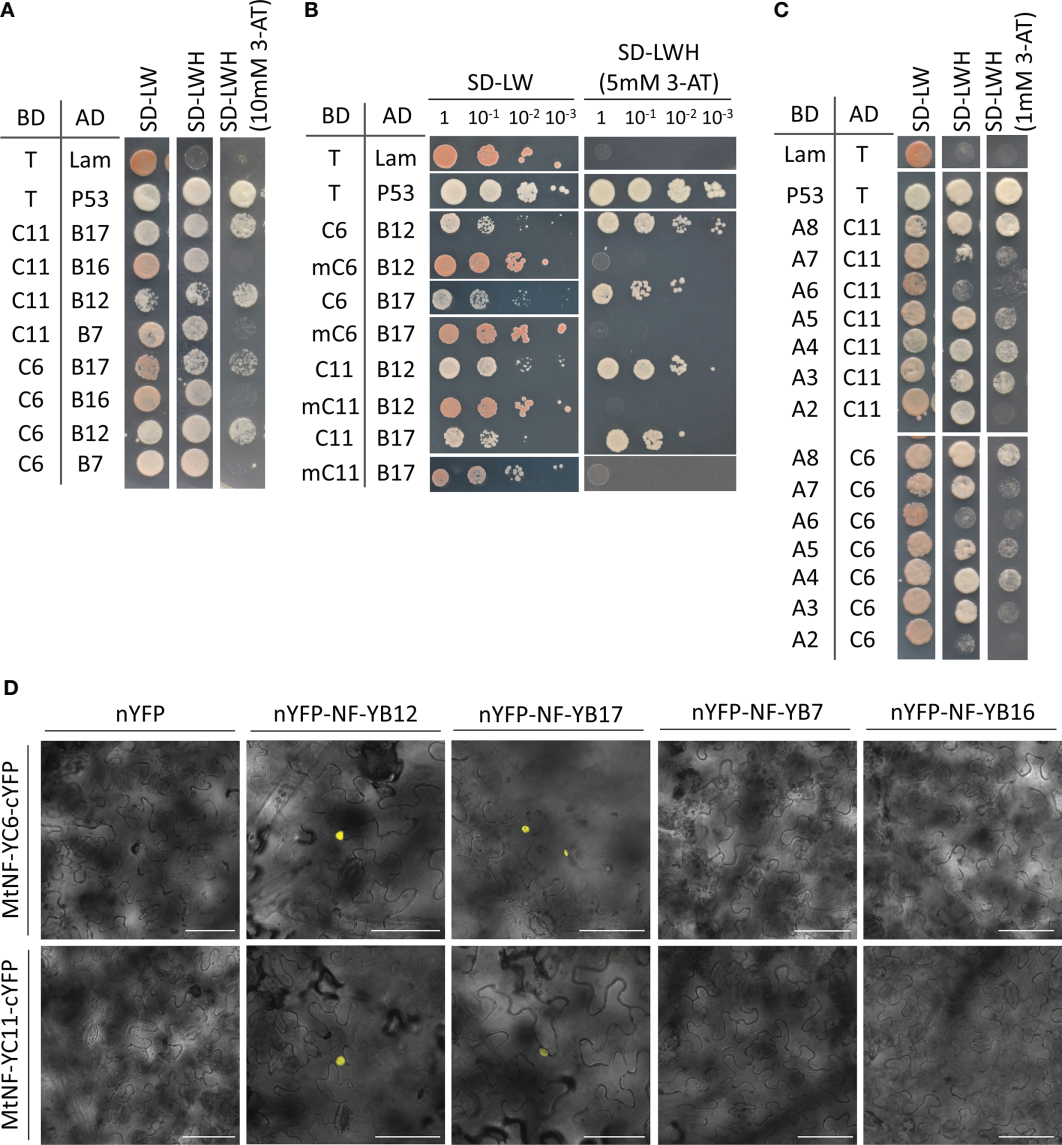
Identification of the interacting partners of MtNF-YC6 and MtNF-YC11 using yeast two-hybrid and BiFC assays. **(A)** Interaction of MtNF-YC6 (C6) or MtNF-YC11 (C11) with four different MtNF-YBs (B7, B12, B16, and B17). **(B)** Interaction of wild type or mutated MtNF-YC6 (mC6) and MtNF-YC11 (mC11) either with MtNF-YB12 (B12) or MtNF-YB17 (B17). **(C)** Interaction of MtNF-YC6 or MtNF-YC11 with seven different MtNF-YAs. Interactions of T with p53 and Lam were used as a positive and negative control, respectively. BD, Gal4 binding domain. AD, Gal4 activation domain. **(D)** Co-expressing C-terminal YFP (cYFP)-tagged MtNF-YC6 or MtNF-YC11 either with N-terminal YFP (nYFP)-tagged MtNF-YB7, B12, B16 or B17 in *N. benthamiana* leaves. The images were the overlay of bright field and YFP. Bar = 75 μm.

To identify the components in the MtNF-YC6-or MtNF-YC11-containing transcriptional complex, we first performed yeast two-hybrid assays to test the interaction between different subunits. In the *Medicago* genome, there are 19 NF-YBs, and according to the expression profiles managed by the *M. truncatula* gene expression atlas web server (He et al., 2009), we selected *MtNF-YB7*, *B12*, *B16*, and *B17* which were induced by AMS or highly expressed in mycorrhizal roots (**Supplementary Table 3**) as preys to test their potential for interacting with two AMS-conserved C subunits. We found that both MtNF-YB12 and MtNF-YB17 could interact either with MtNF-YC6 or MtNF-YC11, but MtNF-YB6 and MtNF-UB17 could not interact (**Figure 1A**). A study on animal NF-YC pointed out an isoleucine and an aspartic acid within histone fold motif as essential residues for the interaction with B subunits (Kim et al., 1996). Thus, we generated mutated MtNF-YC6 (mC6) and MtNF-YC11 (mC11) (**Supplementary Figure 2**). In yeast the mutation at these two essential residues impeded the interaction with MtNF-YB12 and MtNF-YB17 (**Figure 1B**), supporting the importance of these two conserved residues.

To further verify the interaction *in planta*, we examined the subcellular localization of these NF-Y subunits. When transiently expressed in the leaf epidermal cells of *N. benthamiana*, both GFP-tagged MtNF-YC6 and MtNF-YC11 were observed in the cytoplasm and nuclei (**Supplementary Figure 3A**). Similarly, YFP-tagged MtNF-YB12 and MtNF-YB17 were also detected both in the cytoplasm and nuclei (**Supplementary Figure 3B**). Interestingly, when co-expressing CFP-MtNFYC6/C11 with either YFP-MtNF-YB12 or YFP-MtNF-YB17, both CFP and YFP signals were predominantly colocalized in nuclei except some weak signals of YFP-MtNF-YB12 in cytoplasm (**Supplementary Figures 3C, D**). Next, we performed a BiFC assay to further confirm the direct interaction *in planta*. Co-expression of C-terminal YFP (cYFP)-tagged MtNF-YC6/C11 (MtNF-YC6/C11-cYFP) either with N-terminal YFP (nYFP)-tagged MtNF-YB12 (nYFP-MtNF-YB12) or MtNF-YB17 (nYFP-MtNF-YB17) produced YFP signals exclusively in nuclei while co-expression of MtNF-YC6/C11 either with empty vector control or MtNF-YB7 or MtNF-YB16 did not give rise to any signal (**Figure 1D**). Likewise, co-expressing MtNF-YC6/C11-nYFP with MtNF-YB12/B17-cYFP or MtNF-YC6/C11-cYFP with MtNF-YB12/B17-nYFP also produced signals in the nucleus (**Supplementary Figure 4**). These results support the direct interaction of MtNF-YC6/C11 and MtNF-YB12/B17, and the formation of heterodimer brought the protein complexes from the cytoplasm to the nucleus.

The NF-YA family has eight members in *Medicago* genome. A yeast two-hybrid assay was performed to identify A subunits, which could associate either with MtNF-YC6 or MtNF-YC11. Concerning MtNF-YA1, which is a known regulator of rhizobium-legume symbiosis expressed specifically in nodules (Laloum et al., 2014; Laporte et al., 2014; Baudin et al., 2015), only seven A subunits were included in the assay. These results show the relatively strong interaction of MtNF-YC6/C11 with MtNF-YA4/A8 and weak interaction with MtNF-YA3/A5 (**Figure 1C**). Due to the failure of YFP-tagged MtNF-YA8 construction for unknown reasons, we only examined the localization of MtNF-YA4. Like other plant NF-YAs, MtNF-YA4 was localized in the nucleus (**Supplementary Figure 5A**). When transiently overexpressing CFP-MtNF-YC6/C11, MtNF-YB12/B17, and YFP-MtNF-YA4 simultaneously in the leaves of *N. benthamiana*, most CFP and YFP signals were exclusively colocalized in the nuclei (**Supplementary Figure 5B**), but co-expressing CFP-MtNF-YC6, MtNF-YB17 and YFP-MtNF-YA4 did not give rise to any fluorescence signal (data not shown). These results supported the interaction between these subunits and their function in the nucleus.

### The responses of NF-Ys to AMS

**Figure 2:**
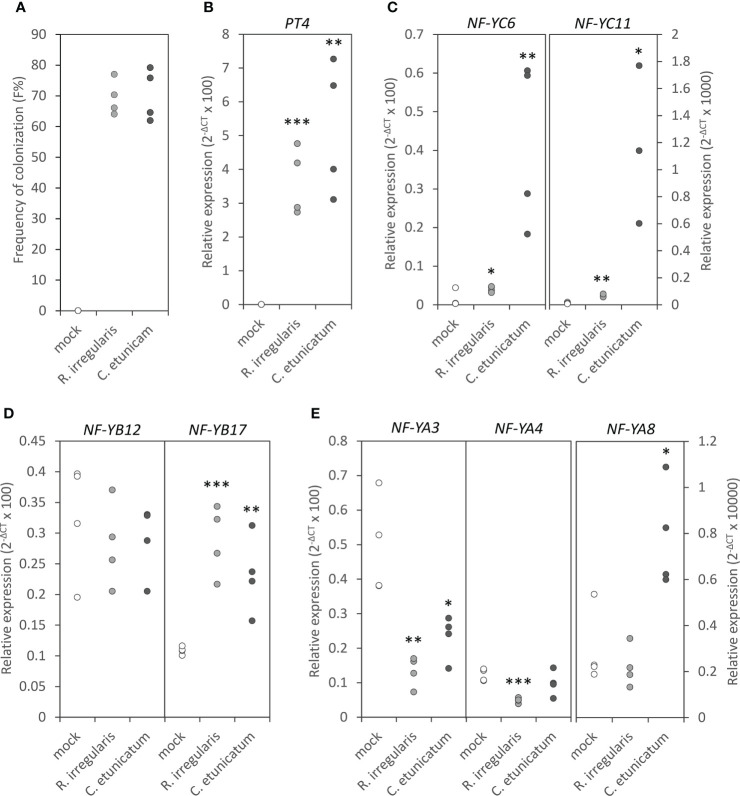
The responses of *MtNF-YC6*, *MtNF-YC11* and their interacting *NF-Y* subunits to AMS. **(A)** The frequency of colonization in AMF-colonized roots. **(B)** The relative expression level of *MtPT4*. **(C-E)** The relative expression level of *MtNF-YC6* and *C11* **(C)**, MtNF-YB12 and B17 **(D)** and *MtNF-YA3*, *A4*, and *A8* **(E)** in mock- and AMF-treated roots. n=4. Student’s t test evaluated the difference between mock- and AMF-treated roots. *p<0.05, **p<0.01, ***p<0.001.

After identifying the components which were able to interact with MtNF-YC6 and MtNF-YC11, we investigated the responses of these components to AMS at transcript levels. First, the expression of *MtNF-YC6, MtNF-YC11*, *MtNF-YB12*, *MtNF-YB17*, *MtNF-YA3*, *MtNF-YA4*, and *MtNF-YA8* in the roots of mock-treated and *C. etunicatum*- or *R. irregularis*-inoculated plants were examined by qRT-PCR. Like the previous report by Hogekamp et al. (2011), we did observe the significant induction of *MtNF-YC6* and *MtNF-YC11* in AMF-treated roots and the expression level of *MtNF-YC11* was much lower than *MtNF-YC6* in mycorrhizal roots. Intriguingly, the induction level of both genes was higher in *C. etunicatum-*colonized roots than in *R. irregularis*-colonized roots (**Figure 2C**) even though the colonization efficiency and the expression of the AMS marker gene, *MtPT4*, in *Medicago* roots by two different AMF species were similar (**Figures 2A, B**). The expression of *MtNF-YB17* was also upregulated by AMS but *MtNF-YB12* was not. Different from the expression pattern of *MtNF-YC6* and *C11*, the transcript levels of both genes were not affected by the difference in AMF species (**Figure 2D**). Unexpectedly, both *MtNF-YA3* and *MtNF-YA4* were downregulated by AMS except in *C. etunicatum*-inoculated roots *MtNF-YA4* was not affected. The expression of *MtNF-YA8* was much lower than *MtNF-YA3* and *MtNF-YA4*, and in contrast, it was upregulated by *C. etunicatum*-inoculation but did not respond to *R. irregularis* colonization (**Figure 2E**).

**Figure 3:**
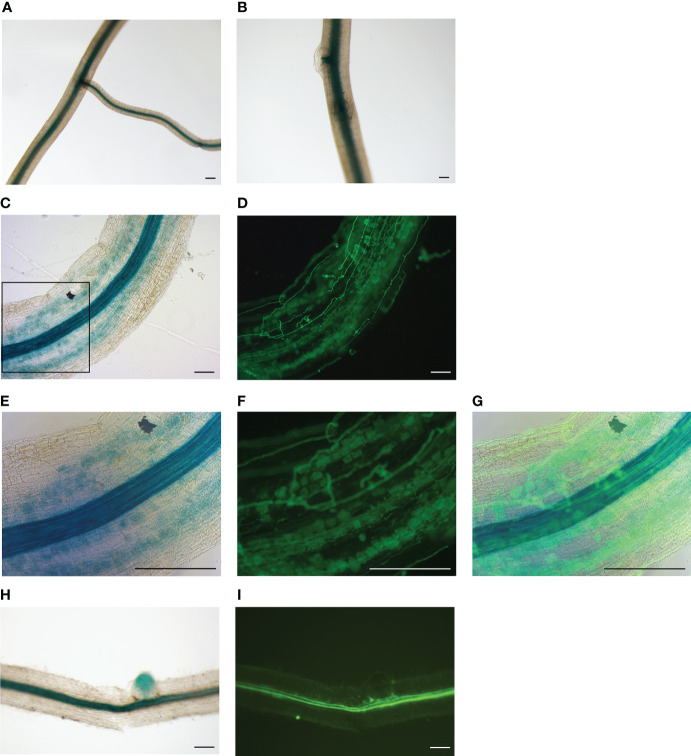
The expression pattern of *MtNF-YB17* in Medicago roots. **(A, B)** GUS staining of roots expressing *MtNF-YB17pro:GUS* from mock-treated root system. **(C-I)** GUS staining of *R. irregularis*-inoculated roots expressing *MtNF-YB17pro:GUS* **(C, E, H)**. Corresponding fluorescence images showing fungal structure stained with WGA at exactly the same region **(D, F, I)**. **(E-G)** Magnification image corresponding to the black rectangle in **(C)**. **(G)** The merged image of bright field and fluorescence. **(H, I)** Non-colonized root fragments expressing *MtNF-YB17pro:GUS* from *R. irregularis*-treated root system. Bar = 200 μm.

The analysis of *MtNF-YB17* transcripts suggested that it was co-activated with *MtNF-YC6*/*C11* by AMS. To determine the expression pattern of *MtNF-YB17* in colonized roots, promoter analysis was used to detect its expression at the tissue level. The GUS activity in composite roots expressing *MtNF-YB17pro:GUS* was analyzed. Blue staining was only observed in the vascular tissues in mock-treated roots (**Figures 3A, B**). But in *R. irregularis*-inoculated roots, strong blue staining was observed in the cortex of colonized regions (**Figures 3C–G**) while in non-colonized regions GUS staining was predominantly observed in vascular tissues (**Figures 3H, I**). In summary, our results suggest that the expression of *MtNF-YB17* was induced by AMS and might function with MtNF-YC6/C11 in the root cortex.

### Functional characterization of MtNF-YC6/C11-containing protein complex during AMS

To illustrate the role of MtNF-YC6/C11-containing protein complexes in AMS, the RNAi technique was used to knock down the expression of either *MtNF-YC6/C11* or *MtNF-YB17* in *Medicago* roots. First, we designed an RNAi construct to knock down *MtNF-YB17* in *R. irregularis*-inoculated roots (**Supplementary Figure 1A**). In RNAi roots, the expression of *MtNF-YC17* was significantly reduced. Although the sequence identity between *MtNF-YB12* and *MtNF-YB17* was not as high as that between *MtNF-YC6* and *C11* (**Supplementary Figure 1**), the expression of *MtNF-YB12* in *MtNF-YB17* RNAi roots was also slightly decreased (**Supplementary Figure 6A**). The frequency of AMF colonization was not affected by the decreased expression of these two B subunits (**Supplementary Figure 6B**). *PT4* and *BCP1*, well-known AMS marker genes, are widely-used symbiotic marker genes. The expression of these two marker genes was also unchanged in RNAi roots (**Supplementary Figure 6C**). We also examined the expression of *MYB1*, a marker gene of arbuscule degeneration that encodes a MYB-type transcription factor (Floss et al., 2017). Similarly, it was not affected by the downregulation of *MtNF-YB17* (**Supplementary Figure 6D**). These results suggest that a few MtNF-YB17 proteins in RNAi roots were sufficient to exert its function or downregulation of *MtNF-YB17* and *MtNF-YB12* was insufficient to affect the AMS process.

**Figure 4:**
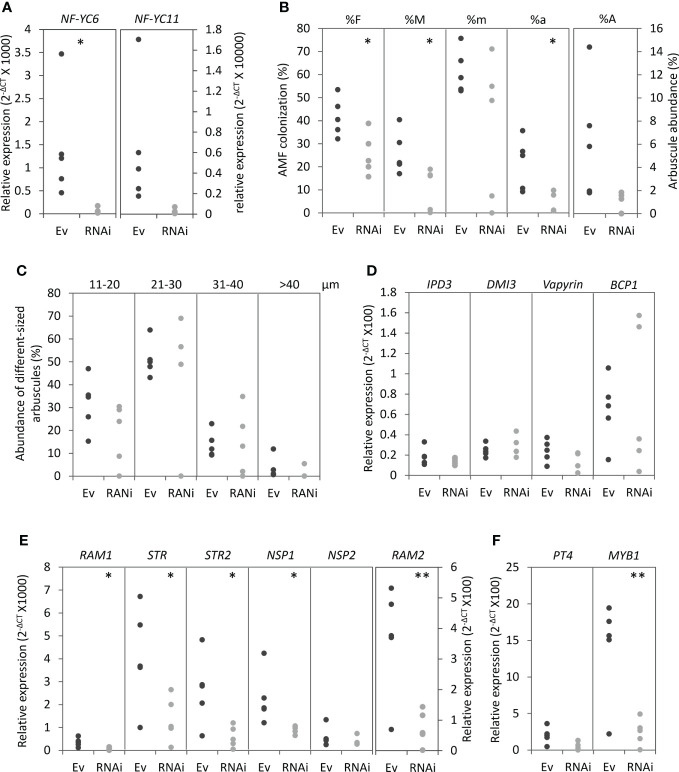
The phenotypic analysis of *MtNF-YC6* and *MtNF-YC11* RNAi roots. **(A)** The relative expression level of *MtNF-YC6* and *C11* in empty vector control (Ev) and RNAi roots (RNAi). **(B, C)** Parameters of AMF colonization **(B)** and the size distribution of arbuscules **(C)** in Ev and RNAi roots. **(D-F)** The relative expression level of AMS marker genes which participated in symbiotic signaling pathway, arbuscule development and arbuscule degeneration. The dark and light gray dots mean Ev and RNAi samples, respectively. n = 5. Student’s t test evaluated the difference between Ev and RNAi. *p<0.05, **p<0.01.

We then investigated the importance of MtNF-YC6 and MtNF-YC11. High sequence similarity between these two genes implied the possibility of functional gene redundancy. Thus, we selected a 300 bp-conserved sequence in the coding sequence to design an RNAi construct (**Supplementary Figure 1B**) to simultaneously knock down both genes. Due to the higher induction level of these two genes in *C. etunicatum-* than in *R. irregularis-*colonized roots, we used *C. etunicatum* as the inoculants to test the roles of these two C subunits in AMS. In RNAi roots, *MtNF-YC6* was significantly downregulated, and *MtNF-YC11* was also decreased by RNAi but not statistically significant (**Figure 4A**). The expression of other members in *MtNF-YC* family was also examined except *MtNF-YC3* and *MtNF-YC10* which do not express in roots. None of family members was affected in RNAi roots (**Supplementary Figure 7**). On average, the AMF colonization frequency (F%), the intensity of mycorrhization (M%) and the arbuscule richness in the colonized roots (a%) were significantly decreased in RNAi roots, compared to roots transformed with an empty vector (**Figure 4B**) but the size distribution and morphology of arbuscules were not affected (**Figure 4C** and **Supplementary Figure 8**).

**Figure 5:**
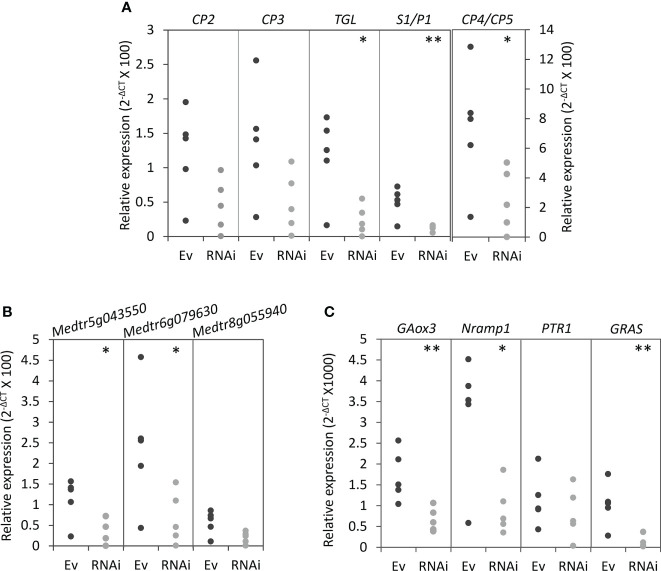
Transcript levels of genes associated with arbuscule degeneration and potential target genes of NF-Y protein complexes in *MtNF-YC6*/*C11* RNAi roots. **(A)** The relative expression level of cysteine protease genes (*CP*s), triacylglycerol lipase (*TGL*) and nuclease S1 (*S1/P1*). **(B)** The relative expression level of chitinase genes. **(C)** The relative expression level of potential target genes of NF-Y complexes. The dark and light gray dots mean Ev and RNAi samples, respectively. n = 5. Student’s t test evaluated the difference between Ev and RNAi. *p<0.05, **p<0.01.

Because silencing *MtNF-YC6* and *MtNF-YC11* resulted in the decrease of colonization frequency and intensity, we further investigated the impacts on the expression of AMS marker genes at different symbiotic processes, including *DMI3* and *IPD3* which participate in the symbiotic signaling pathway (Horváth et al., 2011; Miller et al., 2013) and genes involved in the control of arbuscule development such as *Vapyrin* (Pumplin et al., 2010; Murray et al., 2011), *RAM1* (Gobbato et al., 2012; Park et al., 2015), *RAM2* (Wang et al., 2012; Gobbato et al., 2013), *STR*, *STR2* (Zhang et al., 2010), *NSP1*, *NSP2* (Delaux et al., 2013a; Hofferek et al., 2014), and *BCP1* (Pumplin and Harrison, 2009). Transcripts of *RAM1*, *RAM2*, *STR*, *STR2*, and *NSP1* were significantly reduced in RNAi roots compared to empty vector control, whereas *DMI3*, *IPD3*, *Vapyrin*, and *BCP1* were not affected (**Figures 4D, E**). We also examined *PT4* and *MYB1*, and intriguingly, these two genes were also significantly downregulated in RNAi roots though the reduction level of *PT4* transcripts was not statistically significant (**Figure 4F**). Because of the involvement of PT4 and MYB1 in the arbuscule degeneration, we further analyzed the expression of hydrolase genes associated with this process, including cysteine protease genes (*CP*s), chitinase genes, a triacylglycerol lipase gene (*TGL*) and a S1/P1 type nuclease gene (*S1/P1*) (Floss et al., 2017). Coincidently, the expression of *CP4/CP5*, two of *chitinase* genes, *TGL* and *S1/P1* was significantly reduced in RNAi roots, whereas the transcript levels of *CP2*, *CP3* and the other chitinase genes were slightly decreased in RNAi roots but not significant (**Figures 5A, B**). Although *MtNF-YC6* or *MtNF-YC11* were not simultaneously downregulated in all RNAi roots, the phenotype suggested the involvement of these two genes in regulating the transcriptional program of arbuscule development.

### *In silico* analysis of CCAAT motifs in the promoter regions of AMS-induced genes

Mutations in *Medicago PT4* result in decreased colonization efficiency and a premature arbuscule degeneration phenotype (Harrison et al., 2002; Javot et al., 2007). By analyzing transcriptome associated with arbuscule degeneration, 91 differentially expressed genes (DEG) were identified in *mtpt4* mutant compared to wild type (Floss et al., 2017). The downregulation of *MYB1* and hydrolase genes suggested that MtNF-YC6 and MtNF-YC11 might regulate genes involved in arbuscule degeneration. To understand the importance of NF-Y complexes in the transcriptional regulation at this phase, we performed *in silico* analysis to see the presence of CCAAT-binding motifs in the 2-kbp region upstream of the start codon of 91 DEGs.

We found that 71 DEGs contained at least one CCAAT-binding motif in the promoter regions. Among 71 DEGs, 15 were AMS-conserved genes, including *PT4* and *RAM2*. *MYB1* and several hydrolase genes were also identified as potential downstream targets of NF-Y complexes, such as *CP2*, *CP3*, *CP5*, *TGL*, *S1/P1*, and three chitinase genes which we examined in this study. In addition to *MtPT4*, there were some transporter genes obtained CCAAT-binding motifs in the promoter region, such as *Nramp1*, *PTR1* and *NIP1* (**Table 1**). The location and the number of CCAAT-binding motifs were variable. *MtMYB1* contains four CCAAT-binding motifs in the promoter regions and two of them are located within 200 bp upstream of the start codon. *TGL* had two motifs and one is located 256 bp upstream of the start codon. Both *CP3* and *CP5* contained one motif within 1.5 kbp upstream, and *S1/P1* had three motifs within 500 bp upstream of the start codon (**Table 1**).

**Table 1:** The location of CCAAT motifs were extracted from the 2 kbp upstream region of differentially expressed genes in *mtpt4* mutants.

Gene accession^a^		Location of CCAAT motif	Gene annotation
**Medtr0021s0370**		-1545; -1337; -1193; -1055; -851; -408; -215; -98	signal peptidase 1 (Tau)
Medtr1g011580		-976; -500; -169; -103	gibberellin 3 oxidase
Medtr1g014160		-1721; -1009	tubby C2 protein
**Medtr1g028600**		-622; -560	high affinity inorganic phosphate transporter (MtPT4)
**Medtr1g040500**		-817; -702	glycerol-3-phosphate acyltransferase (RAM2)
Medtr1g061540		-1006; -128	T32M21-140 protein
Medtr1g062970		-261; -171	heparan-alpha-glucosaminide N-acetyltransferase-like protein
Medtr1g090440		-1820; -1666; -1646; -621	C3HC4-type RING zinc finger protein
Medtr1g099310		-166	acidic chitinase
Medtr1g110510		-492; -471; -268	nuclease S1 (S1/P1 nuclease family protein)
**Medtr2g012790**		-901; -687; -380	F-box/kelch-repeat plant protein, putative
**Medtr2g017750**		-791; -284; -50	peptide transporter PTR1 (peptide/nitrate transporter)
Medtr2g055250		-1652; -322; -262; -60	F-box protein
Medtr2g062430		-1324; -1005; -505	DUF946 family protein
Medtr2g075830		-168	cysteine protease (CP1)
**Medtr2g086630**		-1614	germin-like protein
**Medtr2g091215**		-1029; -322; -160	DUF538 family protein
**Medtr2g104800**		-839	DUF4228 domain protein
Medtr3g022830		-1992; -709; -644; -497; -249	GRAS family transcription factor
Medtr3g035700		-1199; -1063; -470	50S ribosomal protein L2, chloroplastic
Medtr3g079190		-1719; -1130; -183	neutral ceramidase (Cer)
Medtr3g082200		-945; -233	subtilisin-like serine protease
Medtr3g088460		-1114; -753	NRAMP metal ion transporter (Nramp1)
Medtr3g109160		-1974; -1669	SAUR-like auxin-responsive family protein
Medtr3g109420		-1019	Hemoglobin
Medtr3g109610		-1697; -630; -294; -265; -23	carotenoid cleavage dioxygenase (MAX4/CCD8a)
Medtr4g005230		-1620; -1555; -1538; -1509; -370; -120	cytochrome P450 superfamily protein
Medtr4g006650		-1997; -1926; -1084; -469	heavy metal-associated transporter
Medtr4g047610		-1836; -1602; -1571; -1489; -1461; -1094; -713	papain family cysteine protease
Medtr4g077180		-961	lipid transfer protein
Medtr4g079770		-1580	cysteine protease (CP5)
Medtr4g079800		-1991; -1066; -381	cysteine protease (CP6)
Medtr4g080700		-1365; -1055	papain family cysteine protease
Medtr4g080730	-1674; -696		cysteine proteinase superfamily protein
Medtr4g091000	-1694; -753; -669; -189; -110		carbohydrate-binding module family 50 protein

a Genes which marked in bold are AMS-conserved genes.

Our data had already shown the significant downregulation of *MYB1*, *PT4*, *RAM2*, *CP5*, *TGL*, *S1/P1* and *chitinases* in *MtNF-YC6/C11* RNAi lines (**Figures 4F**, **5**). We further examined other potential downstream genes, including *Nramp1*, *GA3 hydroxylase* (*GA3ox*), *PTR1* and a gene encoding a GRAS transcription factor (*GRAS*). *Nramp1 GR3ox* and *GRAS* genes were significantly downregulated when *MtNF-YC6/C11* were repressed, but *PTR1*, which encodes a peptide transporter, was not affected (**Figure 5C**). We also analyzed CCAAT motifs in the promoter regions of AMS marker genes we examined in this study and found that all the genes have at least one motif no matter the transcript levels were affected by RNAi construct or not (**Table 2**). These results reveal potential target genes of NF-Y protein complexes, which might be associated with the arbuscule development and degeneration processes. Further validation is required to elucidate the detailed function of NF-Y in the regulatory network.

**Table 2:** The location of CCAAT motifs extracted from 2 kbp upstream region of AMS marker genes included in this study.

Gene accession	Location of CCAAT motif	Gene annotation
Medtr1g105130	-427; -498; -1261; -1461	BCP1
Medtr3g072710	-209; -648	NSP2
Medtr5g026850	-775; -1771	IPD3
Medtr5g030910	-1480; -1551; -1595	STR2
Medtr6g027840	-171; -467; -590; -1215; -1446; -1807	Vapyrin
Medtr7g027190	-298; -815; -1214; -1289	RAM1
Medtr8g020840	-437; -633; -1171	NSP1
Medtr8g043970	-1517; -1542; -1565	DMI3
Medtr8g107450	-580	STR

## Discussion

The involvement of NF-Y protein complexes has been demonstrated in a wide range of processes, such as drought stress response (Nelson et al., 2007), nutrient acquisition (Qu et al., 2015), flower development (Chen et al., 2007) and nodulation process (Laloum et al., 2014; Laporte et al., 2014; Baudin et al., 2015). Two of the C subunits in the *Medicago* NF-Y family, MtNF-YC6 and MtNF-YC11, are specifically induced by AMS and expressed in the epidermal and cortical cells of colonized roots (Hogekamp et al., 2011), but their roles in AMS processes were unclear. Herein, we identify the interacting partners of these two proteins using *in vitro* and *in vivo* interaction assays and reveal their potential function in the transcriptional program of arbuscule development and degeneration.

Using yeast two-hybrid and BiFC assays, we identified MtNF-YB12 and B17 as the interacting partners of MtNF-YC6 and C11 (**Figure 1**). When expressing these proteins individually in *N. benthamiana* epidermal cells, we observed all the fluorescence signals both in the nucleus and cytoplasm (**Supplementary Figures 3A, B**). But co-expressing either one of the B subunits with MtNF-YC6 or C11 led to nuclear-dominant fluorescence signals (**Supplementary Figures 3C, D**). Unlike animal NF-YB, the subcellular localization of plant NF-YB subunits is variable. For example, *Arabidopsis* NF-YB2 is localized predominantly in the nucleus (Cai et al., 2007); AtNF-YB3 and AtNF-YB10 are in the cytoplasm (Liu and Howell, 2010; Hackenberg et al., 2012); while *Medicago* MtNF-YB16, rice OsNF-YB1 and OsNF-YB9 are both in the nucleus and cytoplasm (Baudin et al., 2015; E et al., 2018). Similarly, nuclear- and nucleocytoplasmic-localized NF-YCs were observed *in planta* (Hackenberg et al., 2012; Baudin et al., 2015; E et al., 2018). For cytoplasmic-localized B and C subunits of the NF-Y family, heterodimer formation usually results in the translocation of NF-YB/C heterodimers to the nucleus (Liu and Howell, 2010; Hackenberg et al., 2012; Baudin et al., 2015; E et al., 2018). In this study, we also observed the change of subcellular location from the cytoplasm to the nucleus when co-expressing MtNF-YC6/C11 either with MtNF-YB12 or MtNF-YB17. Meanwhile, no interaction was observed when co-expressing these two C subunits with MtNF-YB7 or MtNF-YB16 in the BiFC assay (**Figure 1**), implying the specificity of protein-protein interaction. However, the interaction specificity between B and C subunits in the NF-Y family remains subject to debate. Among all the NF-YB/C combinations in *Arabidopsis*, most of the possible interactions can be verified by yeast two-hybrid assays in Calvenzani et al. (2012), but only 31% of B/C heterodimerization can be observed in the study by Hackenberg et al. (2012). In the yeast two-hybrid system, the candidate genes are overexpressed in the cells which increases the possibility of protein-protein interaction. *In planta*, the dimerization depends on the abundance and the expression pattern of proteins. It has been shown that *MtNF-YC6* and *C11* were activated in arbuscule-containing cortical cells and adjacent cells (Hogekamp et al., 2011), and in this study, we observed the strong promoter activity of *MtNF-YB17* in the cortex of colonized root fragments (**Figure 3**), supporting that MtNF-YB17 might function with these two C subunits in colonized roots.

Among the four B subunits we chose for the protein-protein interaction test, the amino acid sequence identity between MtNF-YB12 and B17 is the highest (77%; **Supplementary Table 4**). Moreover, the phylogenetic analysis found that these two proteins were derived from the same legume-specific gene duplication events while MtNF-YB7 and MtNF-YB16 were in different subgroups (Laloum et al., 2013), implying that MtNF-YB12 and MtNF-YB17 might have similar functions. It has been shown that MtNF-YB16 forms heterotrimeric complexes with MtNF-YC1/C2 and MtNF-YA1/A2 to control nodule formation (Baudin et al., 2015) but the function of MtNF-YB7, B12, and B17 has not yet been characterized. Our study showed that only *MtNF-YB17* was upregulated in roots either inoculated with *R. irregularis* or *C. etunicatum* relative to mock-treated roots (**Figure 2D**), further supporting our speculation about the involvement of MtNF-YB17 in the control of AMS processes. Although *MtNF-YB12* did not respond to AMS at transcript level, we cannot exclude its role in symbiosis without examining the expression at the protein and tissue levels. In RNAi roots, the expression level of *MtNF-YB17* and *MtNF-YB12* was reduced to 1/5 and 1/2, respectively, relative to the empty vector control; however, neither AMF colonization efficiency nor the expression of AMS marker genes was affected (**Supplementary Figure 6**). The high expression level of *MtNF-YB16* in nodules and the interaction with NF-Y subunits involved in controlling nodule development supported its role in nitrogen-fixation symbiosis. But reducing its expression or even the other three close-related B subunits in roots did not affect symbiotic processes (Baudin et al., 2015). According to expression profiles shown in the *M. truncatula* gene atlas (Carrere et al., 2021), *MtNF-YB12* and *B17* are also expressed in other tissues and are induced by other kinds of treatments, while *MtNF-YC6* and *C11* are more specifically upregulated by AMS, implying that the B subunits might function in several physiological or developmental processes, and other B subunits might function redundantly to complement the partial loss of *MtNF-YB12* and *B17*. Our results suggest that, due to the functional redundancy and relatively low interaction specificity, it might not be enough to influence physiological processes by reducing the expression of one or a few close-related genes in the same family.

The transcripts and expression pattern of *MtNF-YC6*/*C11* are shown in this study and in Hogekamp et al. (2011) suggested their involvement in regulating all stages of AMS. Reducing their expression by RNAi decreased the frequency of AMF colonization, intensity of mycorrization and arbuscule abundance but the size distribution of arbuscule was not affected (**Figures 4B, C**). At molecular level, the expression of genes involved in arbuscule development was downregulated in RNAi roots coincided with the symbiotic phenotype. But the transcript levels of *DMI3* and *IPD3* which function in the initiation of cell redifferentiation and the induction of downstream symbiotic-related genes (Takeda et al., 2012; Takeda et al., 2015) were not changed by RNAi construct (**Figures 4D, E**). Hogekamp et al. (2011) concluded that MtNF-YC6 and C11 function at all AMS stages, but interestingly, in later symbiotic stages the promoter activities of *MtNF-YC6* and *C11* were restricted in cells containing arbuscules or adjacent cortical cells. In this study, the composite roots were harvested at later AMS stages. It is possible that MtNF-YC6 and C11 play more important roles in regulating gene expression in inner cortical cells rather than in epidermal cells, thus genes involved in early symbiotic signaling was not significantly affected by RNAi construct. Previous studies showed that mutation at genes involved in controlling arbuscule development such as *RAM1*, *RAM2*, *STR* and *STR2* resulted in the reduction of colonization and the malformed arbuscules (Zhang et al., 2010; Gobbato et al., 2012; Wang et al., 2012; Delaux et al., 2013a; Gobbato et al., 2013; Hofferek et al., 2014; Park et al., 2015). Although these genes were significantly downregulated by *MtNF-YC6/C11* RNAi construct, the proteins which remain in the roots might still function properly to promote arbuscule development. Thus, we did not observe the significant change of size distribution of arbuscules (**Figure 4C**). Besides, the RNAi construct was designed to silence both genes simultaneously, but the level of reduction of these two genes was different between composite roots. Because of the potential of gene functional redundancy and linkage between two genes on chromosome 2, gene editing technique will be useful for generating double mutants for future functional studies.

In addition to genes involved in arbuscule development, transcripts of genes associated with arbuscule degeneration were also reduced in *MtNF-YC6*/*C11* RNAi roots. The knowledge is about the regulation of arbuscule degeneration is limited. Loss-of PT4 function or overexpressing *MYB1* enhanced hydrolase gene expression, resulting in premature arbuscule degeneration (Javot et al., 2007; Floss et al., 2017). In contrast, reducing *MYB1* transcripts only did not affect the arbuscular phenotype and the expression of AMS marker gene, *MtPT4*, and the fungal *α-tubulin* gene, but the transcript levels of hydrolase genes were significantly decreased (Floss et al., 2017), which was similar to what we observed in *MtNF-YC6*/*C11* RNAi roots. *In silico* analysis showed that *PT4*, *MYB1*, and hydrolase genes had at least one CCAAT-binding motif (**Table 1**). The downregulation of *MYB1*, *CP4/CP5*, *TGL*, *S1/P1* and chitinase genes in *MtNF-YC6/C11* RNAi roots (**Figures 4F**, **5**) implied that NF-Y complexes might directly or indirectly regulate these genes at the transcript levels. Although no premature arbuscule degeneration was observed in *MtNF-YC6*/*C11* RNAi roots, considering the transcript levels of *MYB1* and hydrolase genes shown in this study and the phenotype of *myb1* single mutant as described by Floss et al. (2017), we speculated that MtNF-YC6/C11-containing protein complexes participated in regulating the transcriptional program of arbuscule degeneration. But we still cannot exclude the possibility that the downregulation of *MYB1* and hydrolase genes in RNAi roots was due to the decrease of AMF colonization and arbuscule richness. Further validation by chromatin immunoprecipitation and genetic studies may give more insights into the role of the NF-Y complex in the regulatory network of arbuscule degeneration.

*In silico* analysis showed that all the AMS marker genes and the potential target genes we examined in this study have at least one CCAAT motif, but the transcript levels of these genes were not always well-correlated to the expression of *MtNF-YC6*/*C11.* It is possible that *MtNF-YC6*/*C11*-containing protein complexes are not the key regulators of these genes, or the regulation by other factors compensates the role of NF-Ys in RNAi roots. Another possible reason is the variation of NF-Y binding affinity on different genes. It has been shown that the epigenetic markers on the CCAAT motifs, the distance between two motifs and the flanking sequences affect the NF-Y binding affinity *in vivo.* For example, methylation on the motifs interferes the formation of protein-DNA complex (Bi et al., 1997). The sequence logo analysis showed that C-A-G was moderately prevalent in the downstream of CCAAT motifs of validated target genes (Zambelli and Pavesi, 2017). Further investigation will be required to decipher the binding specificity and affinity of NF-Ys.

Another interesting thing is the responses of *MtNF-YA3*, *MtNF-YA4*, and *MtNF-YA8* to AMS. These A subunits were able to interact either with MtNF-YC6 or MtNF-YC11 in yeast, but the transcript levels of *MtNF-YA3* and *MtNF-YA4* were reduced by AMS while *MtNF-YA8* was not affected or upregulated by *C. etunicatum* symbiosis (**Figure 2E**). The expression level of *MtNF-YA8* was relatively low compared to *MtNF-YA3* and *A4*, and the response was the same as the data shown in the *M. truncatula* gene expression atlas (**Supplementary Table 3**). Transcriptomic analysis showed that *MtNF-YA8* was induced more than 4 folds at 48 h after rhizobium inoculation (Schiessl et al., 2019), suggesting that it might play a more important role in rhizobium-legume symbiosis than in AMS. The opposite expression pattern of *MtNF-YA3* and *A4* compared to *MtNF-YC6/C11* and *MtNF-YB17* implied that they might function differently during AMS. Arabidopsis NF-YB9 acts as an enhancer of hypocotyl elongation through physical association with PHYTOCROME-INTERACTING FACTOR 4 (PIF4) (Huang et al., 2015), while NF-YCs and NF-YAs function in the opposite. Intriguingly, overexpressing most *AtNF-YA*s led to shortened hypocotyl phenotype under continuous dark conditions, but the responses of these *AtNF-YA*s to dark treatment at the transcript level was different; some were increased while others were decreased or not affected (Myers et al., 2016). These results suggest that different NF-Y compositions might activate or silence physiological processes, and their roles do not always directly coincide with the transcriptional expression. In addition, several reports showed the interaction of NF-YB/NF-YC heterodimer with other transcription factors and the involvement of these heterotrimeric complexes in regulating downstream targets through other *cis*-acting elements (Yamamoto et al., 2009; Kumimoto et al., 2013). In these regulatory mechanisms, NF-YAs may act as a competitor to suppress the formation of trimeric complexes, interfering with the downstream transcriptional regulation (Adrian et al., 2010). In this study, we only examined the expression of three A subunits in response to AMS. Whether these genes play a positive or negative role in symbiosis will need to be addressed in further studies.

*MtNF-YC6* and *MtNF-YC11* are AMS-induced genes. Interestingly, the induction level in roots inoculated with two different AMF species was varied even though the colonization efficiency was similar (**Figure 2C**). We also observed the reduction of *MtNF-YA4* in *R. irregularis*-colonized roots but not in *C. etunicatum*-colonized roots and induction of *MtNF-YA8* only in *C. etunicatum*-colonized roots but not in *R. irregularis*-colonized roots (**Figure 2E**). By comparing the transcript level of AMS-responsive genes in the roots colonized by three different AMF species, Grunwald et al. (2009) found that among common AMS-regulated genes, only a few genes showed overlapping regulation patterns in different AMF-colonized roots, hinting that phylogenetic relationship of AMF species not only determines the fungal morphology and physiology but also affects molecular responses in host plants.

## Conclusion

This study identified the interacting partners of MtNF-YC6 and MtNF-YC11 and potential downstream targets of NF-Y complexes during AMS. The AMF colonization phenotype and AMS marker gene expression demonstrate the involvement of these two genes in the regulation of arbuscule development. This information will help to build up the regulatory network of AMS in the future.

## Data availability statement

The original contributions presented in the study are included in the article/**Supplementary Materials**. Further inquiries can be directed to the corresponding author.

## Author contributions

CD and W-YL performed conceptualization and did methodology. CD, C-JL, C-YH, Y-AC and W-YL did validation and investigation. L-YDL and W-YL performed formal analysis. W-YL did data curation, prepared original draft preparation and contributed to writing—review and editing. W-YL did supervision and funding acquisition. All authors contributed to the article and approved the submitted version.

## Funding

This research was funded by Ministry of Science and Technology, Taiwan (grant number most-108-2313-B-002-023-MY3) and College of Bioresources and Agriculture, NTU, Taiwan (grant number T111T6000009).

## Acknowledgments

We thank Dr. Maria J. Harrison (Boyce Thompson Institute for Plant Research, USA), Dr. Tzyy-Jen Chiou (Academia Sinica, Taiwan) and Dr. Shu-Yi Yang (National Taiwan University, Taiwan) for kindly providing vectors. We thank Dr. Jui-Chang Huang (Tainan District Agricultural Research and Extension Station, Taiwan) for providing AMF inoculants, Dr. Chang-Lin Chen (National Chung Hsing University, Taiwan) for helping composite root generation, and Dr. Yu-Chang Tsai (National Taiwan University, Taiwan) for assistance in fluorescence light microscopy.

## Conflict of interest

The authors declare that the research was conducted in the absence of any commercial or financial relationships that could be construed as a potential conflict of interest.

## Publisher’s note

All claims expressed in this article are solely those of the authors and do not necessarily represent those of their affiliated organizations, or those of the publisher, the editors and the reviewers. Any product that may be evaluated in this article, or claim that may be made by its manufacturer, is not guaranteed or endorsed by the publisher.
