# Supplementary material for: MtNF-YC6 and MtNF-YC11 are involved in regulating the transcriptional program of arbuscular mycorrhizal symbiosis
Source: Front Plant Sci. 2022 Sep 28;13:976280. doi: 10.3389/fpls.2022.976280 (PMC9554486; doi:10.3389/fpls.2022.976280)
Supplement: Supplementary file 1 [file DataSheet_1.pdf]

frontiers

Supplementary Material

Supplementary Table 1. The list of primers used for plasmid construction.

Constructs	Primer sequence
pDONR221-MtNF-YC6	Forward: GGGGACAAGTTTGTACAAAAAAGCAGGCTTCATGAGACAAGCAGGTGCAT<br>Reverse: GGGGACCACTTTGTACAAGAAAGCTGGGTCCCTTCGTTCAGCTGAATTAGAA
pDONR221-MtNF-YC11	Forward: GGGGACAAGTTTGTACAAAAAAGCAGGCTTCATGAGACAAGCAGGTGCAT<br>Reverse: GGGGACCACTTTGTACAAGAAAGCTGGGTCTCATATACTTTCCATTTGCATG
pDONR221-MtNF-YB7	Forward: GGGGACAAGTTTGTACAAAAAAGCAGGCTTCATGTCAGGTAATAAGAGAAACC<br>Reverse: GGGGACCACTTTGTACAAGAAAGCTGGGTCTCAACCCTGTAACGTAGG
pDONR221-MtNF-YB12	Forward: GGGGACAAGTTTGTACAAAAAAGCAGGCTTCATGGCGGACTCTGACAAC<br>Reverse: GGGGACCACTTTGTACAAGAAAGCTGGGTCCTATCTGGGCCTACCAGTA
pDONR221-MtNF-YB16	Forward: GGGGACAAGTTTGTACAAAAAAGCAGGCTTCATGATGGATAACAACGTTGG<br>Reverse: GGGGACCACTTTGTACAAGAAAGCTGGGTCTTCCTAGTTGTGGAATCATG
pDONR221-MtNF-YB17	Forward: GGGGACAAGTTTGTACAAAAAAGCAGGCTTCATGGCTGATTCAGACAATG<br>Reverse: GGGGACCACTTTGTACAAGAAAGCTGGGTCACCATATCTACCTGGGTCTA
pDONR221-MtNF-YC6/C11 RNAi	Forward: GGGGACAAGTTTGTACAAAAAAGCAGGCTTCATGAGACAAGCAGGTGCAT<br>Reverse: GGGGACCACTTTGTACAAGAAAGCTGGGTCAGAAACCAAAGTGATCAAAAAATCAAA
pDONR221-MtNF-YB17 RNAi	Forward: GGGGACAAGTTTGTACAAAAAAGCAGGCTTCTGAAGCTTCCGATAAGTGTCA<br>Reverse: GGGGACCACTTTGTACAAGAAAGCTGGGTCACCATATCTACCTGGGTCTA
pDONR221-MtNF-YB17pro	Forward: GGGGACAAGTTTGTACAAAAAAGCAGGCTTCATTGCAGTGAAAGGACCCCA<br>Reverse: GGGGACCACTTTGTACAAGAAAGCTGGGTCCGTCACTACTCCGAATCGTT
TOPO-MtNF-YC6	Forward: ATGAGACAAGCAGGTGCATATTCA<br>Reverse: GGGGACCACTTTGTACAAGAAAGCTGGGTCCCTTCGTTCAGCTGAATTAGAA
TOPO-MtNF-YC11	Forward: ATGAGACAAGCAGGTGCATATTCA<br>Reverse: GGGGACCACTTTGTACAAGAAAGCTGGGTCTCATATACTTTCCATTTGCATG
TOPO-MtNF-YA2	Forward: ATGGCTATGCAAACTGTTTATCTGA<br>Reverse: TCAAGCTTTAAGGTTACAGCAGTT
TOPO-MtNF-YA3	Forward: CCCAAAGGTTACATGAAGCCT<br>Reverse: TCACCGGACAACAGAAGC
TOPO-MtNF-YA4	Forward: ATGAAGTGCTTATGCGAGAAAG<br>Reverse: TCACATGCGGACGGATAG
TOPO-MtNF-YA5	Forward: ATGCAACCCAAGTCCGAAA<br>Reverse: CTACTGAATAGCAAGACGCCT
TOPO-MtNF-YA6	Forward: ATGACTTCTTCCACACATGATCC<br>Reverse: TCAAGGTTGTTTATCAGATTGTGC
TOPO-MtNF-YA7	Forward: ATGCCAGGGAAACCAGAAA<br>Reverse: CACCTCGTGCGGTATGAG
TOPO-MtNF-YA8	Forward: ATGAAGCACTCATGGCCATG<br>Reverse: TCACATAAGGACAGATAGTCGGT

Supplementary Table 2. The list of primers used for qRT-PCR.

Genes	Primer sequence	Reference
MtNF-YC1 (Medtr3g072710)	Forward: GCAGCAGCAATCACAGGAAC<br>Reverse: AATTCCATCAGCAGGCCCTC	
MtNF-YC2 (Medtr1g082660)	Forward: GGAGTTAACCCTGCGCTCAT<br>Reverse: ACGAGGCACAATGTCAACCA	
MtNF-YC4 (IMGA_contig_49832_3.1)	Forward: AAGATGGGCTTGGAATGGCA<br>Reverse: ATGAAAGCCACAGGAGGTCG	
MtNF-YC5 (Medtr5g088760)	Forward: TGTGCGAGAGTCCGATGAAG<br>Reverse: TGGGATAGGAGTTGGTGTGGA	[1]
MtNF-YC6 (Medtr2g081600)	Forward: ACCACTGTCATGCAAATGGAA<br>Reverse: TCAGCTG AATTAGAAGTGACGA	
MtNF-YC7 (Medtr4g059710)	Forward: TTAGCGGAAGATGCTTATGGTTG<br>Reverse: CAGGAACAAAGTCGGACAGAAA	[1]
MtNF-YC8 (Medtr3g012030)	Forward: GCCCCATTGCCTGAACCT<br>Reverse: CCTCATCATAATCTTCCTCATCC	[1]
MtNF-YC9 (Medtr3g085430)	Forward: CGGTGAAGAGAAACCGTCCA<br>Reverse: CGAGTACCAACCGAAACGGA	[1]
MtNF-YC11 (Medtr2g081630)	Forward: CGTGAAAATGATATCAGGGGTTG<br>Reverse: AGAGTGGTGGCATCAGTACTAG	
MtNF-YB12 (Medtr3g058980)	Forward: CGCAATCCGAGTTGAATCCG<br>Reverse: CTCGTGGTGACATCTCGCTT	
MtNF-YB17 (Medtr5g095740)	Forward: GCGAAGATCTCGAAAGATGC<br>Reverse: CATCGCCCAAAGTAGATCGT	
MtNF-YA3 (Medtr2g041090)	Forward: ATGGCTCAGACTCATGCTCA<br>Reverse: GCAACTAACGAACCAGCGAA	
MtNF-YA4 (Medtr2g099490)	Forward: TCCAGGCTACAATGGCCTAC<br>Reverse: GACATGATGCTATAAGCCTGTAACT	
MtNF-YA8 (Medtr8g019540)	Forward: CGGTGGAAAGAGTACGGAAG<br>Reverse: GGAATGCAACAGGAGCAAGT	
MtRAM1 (Medtr7g027190)	Forward: AAGCCATTTTCGAGGCGTTT<br>Reverse: CGTTAAGCATCGTCCGGTTT	[2]
MtRAM2 (Medtr1g040500)	Forward: AAACCTGGAATACTTGTTGGAGA<br>Reverse: TGTTACCTTTGGCTTTGCTG	
MtIPD3 (Medtr5g026850)	Forward: GCGCTCAAGAAAAATGGCTGAAGC<br>Reverse: GCTTTAGTGATCGAACTTCCTTCTCAAGG	[3]
MtVapyrin (Medtr6g027840)	Forward: GGAGGTGAGGACAATCCAAA<br>Reverse: GTCCTGATTCAGCAGCACAA	[4]
MtDMI3 (Medtr8g043970)	Forward: ACCGTGATGGAACAGTTGACA<br>Reverse: TCTTTGCTGATGCAGCCTGA	[3]
MtSTR (Medtr8g107450)	Forward: TTCCAATGATGCAGTCCCA<br>Reverse: TGGTTATGACTGCAAATGTGAG	[5]
MtSTR2 (Medtr5g030910)	Forward: GCAAGTGGGAGTCTTAAAGGA<br>Reverse: GCCCTAATCTGAAATCAGCAG	[5]
MtNSP1 (Medtr8g020840)	Forward: GCGATTTCGCCACTGGATTC<br>Reverse: CAGCCTCGCCTTCCATCATT	[3]
MtNSP2 (Medtr3g072710)	Forward: GCTTCCAACAACAACGGTCC<br>Reverse: TAATCGCCTGCCGGTTTCTT	[3]

Supplementary Table 2. The list of primers used for qRT-PCR (continued).

Genes	Primer sequence	Reference
MtPT4 (Medtr1g028600)	Forward: GACACGAGGCGCTTTCATAGCAGC<br>Reverse: GTCATCGCAGCTGGAACAGCACCG	[2]
MtBCP1 (Medtr1g105130)	Forward: TCCATGGTTTTGCTTTCCTC<br>Reverse: CGGTGGAAAGTGCTTCATTT	[2]
MtMyb1 (Medtr7g068600*)*	Forward: TAAGAGAGTTGATGATGATGTTC<br>Reverse: GATGAGTGATTCTGTTGAACC	[6]
MtPTR1 (Medtr2g017750)	Forward: GAACCAAAGCTGCATTGCCA<br>Reverse: CCCATGCAAACAGCAAGCAA	
Nramp1 (Medtr3g088460)	Forward: TGTTGGGACGTTCAAGCTCA<br>Reverse: TGGTGCTATGGCAACAATCCT	
CP2 (Medtr5g022560)	Forward: TGCTTACTAATCAACCTCAAAGCA<br>Reverse: TAGCCCACAATCCCAAGCAG	
CP3 (Medtr4g107930)	Forward: AACAATGATGCCAATAACAAGC<br>Reverse: GGAGCACATATGACCCTTGA	[6]
CP4/CP5 (Medtr4g079470 & Medtr4g079770)	Forward: CCAACAATGAGCAGGCACT<br>Reverse: GACACCATGGTCCAACTCAG	[6]
Chitinase (Medtr5g043550)	Forward: ATCCTCTCAGGACGCGAAAC<br>Reverse: AGACGGAGATTGTCCTCCCA	
Chitinase (Medtr6g079630)	Forward: GGCATGTCAGAGGTTGAAGAG<br>Reverse: GCCAAAAGTGCTTGTGATTG	[6]
Chitinase (Medtr8g055940)	Forward: CGTGGAAGCAGTGGACATCA<br>Reverse: TCTGCGGAAATAAAACCGCT	
TGL (Medtr7g081050)	Forward: CAACTTACGACCTGACCAAA<br>Reverse: TGATCTTGGAGGTCATTGAG	[6]
S1/P1 (Medtr1g110510*)*	Forward: GATGCCCCTGAAGATTCTGT<br>Reverse: AAATACGATTGAGGGTTGCTG	[6]
GA3ox (Medtr1g011580)	Forward: CTACCCATGTTGTCCCGACC<br>Reverse: TGGATGAGGGTCCACAGGAA	
GRAS (Medtr7g069740)	Forward: GCATAGATGGTGGAAGATTGAGC<br>Reverse: ATGTTCACTCCTAATTTCCTAGACT	
MtEF1-α (Medtr6g021805)	Forward: TGACAGGCGATCTGGTAAGG<br>Reverse: TCAGCGAAGGTCTCAACCAC	[2]

[1] Potsenkovskaia, E.; Tvorogova, V.; Yakovleva, D.; Zlydneva, N.; Lutova, L. Novel NF-Y genes expressed during somatic embryogenesis in *Medicago truncatula*. *Plant Gene* **2022**, *31*, 100364.

[2] Park, H.J.; Floss, D.S.; Levesque-Tremblay, V.; Bravo, A.; Harrison, M.J. Hyphal branching during arbuscule development requires *Reduced Arbuscular Mycorrhiza1*. *Plant Physiol* **2015**, *169*, 2774-2788.

[3] Floss, D.S.; Levy, J.G.; Levesque-Tremblay, V.; Pumplin, N.; Harrison, M.J. DELLA proteins regulate arbuscule formation in arbuscular mycorrhizal symbiosis. *Proc Natl Acad Sci U S A* **2013**, *110*, E5025-5034.

[4] Lindsay, P.L.; Williams, B.N.; MacLean, A.; Harrison, M.J. A Phosphate-Dependent Requirement for Transcription Factors IPD3 and IPD3L During Arbuscular Mycorrhizal Symbiosis in *Medicago truncatula*. *Mol Plant Microbe Interact* **2019**, *32*, 1277-1290.

[5] Zhang, Q.; Blaylock, L.A.; Harrison, M.J. Two *Medicago truncatula* Half-ABC Transporters Are Essential for Arbuscule Development in Arbuscular Mycorrhizal Symbiosis. *Plant Cell* **2010**, *22*, 1483-1497.

[6] Floss, D.S.; Gomez, S.K.; Park, H.J.; MacLean, A.M.; Muller, L.M.; Bhattarai, K.K.; Levesque-Tremblay, V.; Maldonado-Mendoza, I.E.; Harrison, M.J. A transcriptional program for arbuscule degeneration during AM symbiosis is regulated by MYB1. *Curr Biol* **2017**, *27*, 1206-1212.

Supplementary Table 3. The expression levels of *MtNF-Y*s in AMF colonized roots.

Gene symbol	Accession number	Log_2 (Ri/-P) [a]	LCM root arbuscular [b]	LCM root adjacent [c]	LCM root cortical [d]
MtNF-YA1	Medtr1g056530	-1.34	26.15	15.94	50.6
MtNF-YA2	Medtr7g10645	-2.12	34.55	212.66	98.24
MtNF-YA4	Medtr2g099490	-1.07	41.12	172.75	27.99
MtNF-YA7	Medtr8g037270	-0.13	19.05	58.71	39.14
MtNF-YA3	Medtr2g041090	-1.80	63.45	14.72	35.33
MtNF-YA6	Medtr2g030170	-0.20	167.82	78.46	174.61
MtNF-YA5	Medtr3g061510	-0.65	70.19	247.55	26.12
MtNF-YA8	Medtr8g019540	-0.37	16.42	7.88	9.37
MtNF-YB1	Medtr2g056000	-0.31	40.32	55.58	71.33
MtNF-YB5	Medtr4g112380	0.03	109.71	496.91	155.29
MtNF-YB4	Medtr4g052950	0.29	17.76	54.85	24.32
MtNF-YB19	Medtr1g029100	NA[e]	NA[e]	NA[e]	NA[e]
MtNF-YB14	Medtr5g095900	NA[e]	NA[e]	NA[e]	NA[e]
MtNF-YB3	Medtr4g133952	-0.31	31.4	10.46	12.15
MtNF-YB10	Medtr1g039040	-0.32	70.71	20.36	24.55
MtNF-YB12	Medtr3g058980	-0.25	4123.9	4819.2	3057.73
MtNF-YB8	Medtr4g133938	0.15	31.68	129.37	11.56
MtNF-YB6	Medtr1g088860	-1.06	68.07	116.33	35.13
MtNF-YB11	Medtr1g083070	0.55	14.07	30.77	39.07
MtNF-YB16	Medtr4g119500	2.40[f]	NA[e]	NA[e]	NA[e]
MtNF-YB18	Medtr0392s0020	NA[e]	NA[e]	NA[e]	NA[e]
MtNF-YB15	Medtr8g093920	0.09	9.54	10.51	26.43
MtNF-YB17	Medtr5g095740	1.32	29.02	789.51	170.04
MtNF-YB2	Medtr7g100650	-0.17	299.57	303.48	269.24
MtNF-YB9	Medtr1g072790	0.21	36.54	133.45	101.78
MtNF-YB7	Medtr8g091720	3.37	12.63	12.95	18.28
MtNF-YB13	Medtr2g026710	NA[e]	NA[e]	NA[e]	NA[e]
MtNF-YC10	Medtr2g023340	NA[e]	NA[e]	NA[e]	NA[e]
MtNF-YC7	Medtr4g059710	0.38	73.9	256.7	234.98
MtNF-YC9	Medtr3g085430	-0.30	204.41	376.78	124.99
MtNF-YC6	Medtr2g081600	6.55	115.4	99.16	11.34
MtNF-YC11	Medtr2g081630	2.13	6438.19	3408.26	51.72
MtNF-YC4	IMGA_contig_49832_3.1	-0.70	29.21	15.88	45.71
MtNF-YC2	Medtr7g113680	0.10	50.15	170.28	59.65
MtNF-YC1	Medtr1g082660	0.05	274.25	441.1	166.5

Supplementary Table 3. The expression level of *MtNF-Y*s in colonized roots (continued).

Gene symbol	Accession number	Log_2 (Ri/-P) [a]	LCM root arbuscular [b]	LCM root adjacent [c]	LCM root cortical [d]
MtNF-YC3	Medtr3g099180	0.21	27.98	29.05	30.24
MtNF-YC5	Medtr5g088760	-0.28	99.61	57.2	19.16
MtNF-YC8	Medtr3g012030	-0.42	589.38	268.03	945.57

[a] Fold change of gene expression between in *Rhizophagus irregularis*-colonized roots (Ri) and mock-treated roots (-Pi). Microarray data is derived from *Medicago truncatula* Gene Expression Atlas (MtGEA).

[b] Gene expression level in arbuscule-containing cortical cells. Microarray datas are derived from MtGEA.

[c] Gene expression level in cortical cells adjacent to arbuscule-containing cells. Microarray datas are derived from MtGEA.

[d] Gene expression level in non-colonized cortical cells. Microarray datas are derived from MtGEA.

[e] Data is not available.

[f] Data derived from RNA sequencing data reported by Afkhami and Stinchcombe (2016).

Supplementary Table 4. The protein sequence identity and similarity between four MtNF-YBs investigated in this study.

% of Identity (similarity)	MtNF-YB7	MtNF-YB12	MtNF-YB16	MtNF-YB17
MtNF-YB7	100 (100)			
MtNF-YB12	51 (61)	100 (100)		
MtNF-YB16	36 (48)	38 (52)	100 (100)	
MtNF-YB17	53 (62)	77 (81)	39 (55)	100 (100)

(A)

```
MtNF-YB12 ATGGCGGACTCTGACAACGACTCCGGCGGACCACACGGCGGAGGATCAAACGCTCACGGA 60
MtNF-YB17 ATGGC------------TGATTCAGACAATGAATCCGGAGGAGCACCGAACGCCGGCAAC 48
          *****             ** ** * *     *  *** **** * * *****   *

MtNF-YB12 AGCGAGATGTCACCACGAGAACAAGACCGATTTCTTCCAATAGCAAACGTAAGCAGGATC 120
MtNF-YB17 AGCGAGTTATCACCACGAGAACAAGACCGATTTTTACCAATAGCAAACGTGAGCAGAATA 108
          ****** * ************************ * ************** ***** **

MtNF-YB12 ATGAAAAAAGCACTACCAGCGAACGCGAAGATCTCAAAAGACGCAAAGGAAACAGTTCAA 180
MtNF-YB17 ATGAAGAAGGCGTTACCAGCAAACGCGAAGATCTCGAAAGATGCTAAGGAAACGGTGCAG 168
          ***** ** **  ******* ************** ***** ** ******** ** **

MtNF-YB12 GAGTGTGTTTCAGAGTTCATCAGCTTCATCACCGGCGAAGCCTCCGATAAGTGTCAACGT 240
MtNF-YB17 GAATGTGTATCGGAGTTCATCAGCTTCATCACCGGTGAAGCTTCCGATAAGTGTCAGAGA 228
          ** ***** ** *********************** ***** **************  *

MtNF-YB12 GAGAAGCGGAAGACGATCAACGGCGATGATTTGCTTTGGGCGATGACGACGTTGGGATTC 300
MtNF-YB17 GAGAAACGAAAGACGATCAACGGCGACGATCTACTTTGGGCGATGACTACGTTAGGATTT 288
          ***** ** ***************** *** * ************** ***** *****

MtNF-YB12 GAGGAGTATGTTGAGCCGTTGAAAGGTTATCTTCAACGGTTTAGGGAGATGGAAGGTGAG 360
MtNF-YB17 GAAGAGTACGTTGAACCGTTGAAGATTTATCTTCAACGATTCAGAGAGATCGAAGGTGAG 348
          ** ***** ***** ********   ************ ** ** ***** *********

MtNF-YB12 AAGACCGTGGGGGCGCGTGATAAAGACGCGCCCCAAGGTTCTGGTAGTGTTACTAATAGT 420
MtNF-YB17 AAAACCGTTGCTGCACGTGATAAAGATGGTGTTGCTCCATCTTCTTCTTCGTCTTCAGTT 408
          ** ***** *  ** *********** *           ***  *  *    **     *

MtNF-YB12 TCTTATGAGAGTGGTGGTTATGGTGGTGGTGGTGGTGTTATGATGCATCAGGGACACGTG 480
MtNF-YB17 TTTGATTATGGTGCTCCTCCTCCTCAAGTTGGATTGATCATGCATCATCAGCATCAGGGA 468
          * * ** *  *** *  *  *  *   *  **     * ***   ******   ** *

MtNF-YB12 TATGGTTCTGGTGGTGGTGGGTTTCATCAAGTTATGGGTAAGGGTGGGCCCGGGTATCCT 540
MtNF-YB17 CACGTGTACGGTTCTGGTGGTTTTCATCAAGTGCCTG------GTGGGCCTGTTTATCCT 522
             *  *  ***  ****** ***********    *      ******* *  ******

MtNF-YB12 GGGCCTGGGTCTAATACTGGTAGGCCCAGATAG 573
MtNF-YB17 AATGCTGGATCTAATGCGGGTAGACCCAGGTAG 555
              **** ****** * ***** ***** ***
```

(B)

```
MtNF-YC6  -------------ATGAGACAAGCAGGTGCATATTCAGGTATACTAAATGGTGGCATAGG 47
MtNF-YC11 ATAAAGAAACAATATGAGACAAGCAGGTGCATATTCAGGTATAGTAAATGGTGGCATAGG 60
                       ****************************** ****************

MtNF-YC6  AAGAACAGGTCCACATTCATTACCATTAGCAAGAATAAAGAAGATAATGAAGAATTCTAG 107
MtNF-YC11 AAGAACAGGTCCACATTCATTACCATTAGCAAGAATAAAGAAGATAATGAAGAATTCTAG 120
          ************************************************************

MtNF-YC6  TGAAGACGTGAAAATGATATCAGGTGTAGCTCCAATTGTTTTCTCAAAAGCTTGTGAACT 167
MtNF-YC11 TGAAGACGTGAAAATGATATCAGGGGTTGCTCCAATTGTTTTCTCAAAAGCTTGTGAACT 180
          ************************ ** ********************************

MtNF-YC6  TTTCATTGAAGAACTTACAAGAAGGTCTTGGATTATGGCTATTGATGCTAAAAGAAGAAC 227
MtNF-YC11 TTTCATTGAAGAACTTACAAGAAGGTCTTGGATTATGGCTATTGATGCTAAAAGAAGAAC 240
          ************************************************************

MtNF-YC6  TTTGAATAAAGAAGATGTTGCTTCTGCTGTTATAGCTACTGATATTTTTGATTTTTTGAT 287
MtNF-YC11 TTTGAATAAAGAAGATGTTGCTTCTGCTGTTATAGCTACTGATATTTTTGATTTTTTGAT 300
          ************************************************************

MtNF-YC6  CACTTTGGTTTCTAATTCTGATTCCACTGATGATACCACTG---TCATGCAAATGGAAAC 344
MtNF-YC11 CACTTTGGTTTCTAATTCTGATTCTAGTACTGATGCCACCACTCTCATGCAAATGGAAAG 360
          ************************ * * **** **** ***************

MtNF-YC6  TATGAATTCTTCTTAATTAGGGTTTATGCTGCAACATATATAATGTAACATCGTCACTTC 404
MtNF-YC11 TATGA------------------------------------------------------- 367
          ***

MtNF-YC6  TAATTCAGCTGAACGAAGG 423
MtNF-YC11 ------------------- 367
```

Supplementary Figure 1. Sequence alignment of *MtNF-YB*s and *MtNF-YC*s. (A) Alignment of coding sequences of *MtNF-YB12* and *MtNF-YB17*. (B) mRNA sequence alignment of *MtNF-YC6* and *MtNF-YC11*. The sequences labeled in bold are the location of primers used for qRT-PCR. The shade region highlights the sequence designed for RNAi construct.

```
HsNF-YC   MSTEGGFGGTSSSDAQQSLQSFWPRVMEEIRNLTVKDFRVQELPLARIKKIMKL-DEDVK 59
MtNF-YC6  MRQAGAYSG--------------------ILNGGIGRTGPHSLPLARIKKIMKNSSEDVK 40
MtNF-YC11 MRQAGAYSG--------------------IVNGGIGRTGPHSLPLARIKKIMKNSSEDVK 40
          *   *.:.*                    * *  :     :.***********  .****

HsNF-YC   MISAEAPVLFAKAAQIFITELTLRAWIHTEDNKRRTLQRNDIAMAITKFDQFDFLIDIVP 119
MtNF-YC6  MISGVAPIVFSKACELFIEELTRRSWIMAIDAKRRTLNKEDVASAVIATDIFDFLITLVS 100
MtNF-YC11 MISGVAPIVFSKACELFIEELTRRSWIMAIDAKRRTLNKEDVASAVIATDIFDFLITLVS 100
          ***. **::*:**.::** *** *:** : * *****:::*:* *:   * ***** :*
```

Supplementary Figure 2. Protein sequence alignment of MtNF-YC6 and MtNF-YC11 with human HsNF-YC. The shade regions are histone fold domains. Black boxes highlight the conserved isoleucine and aspartic acid in NF-YCs.

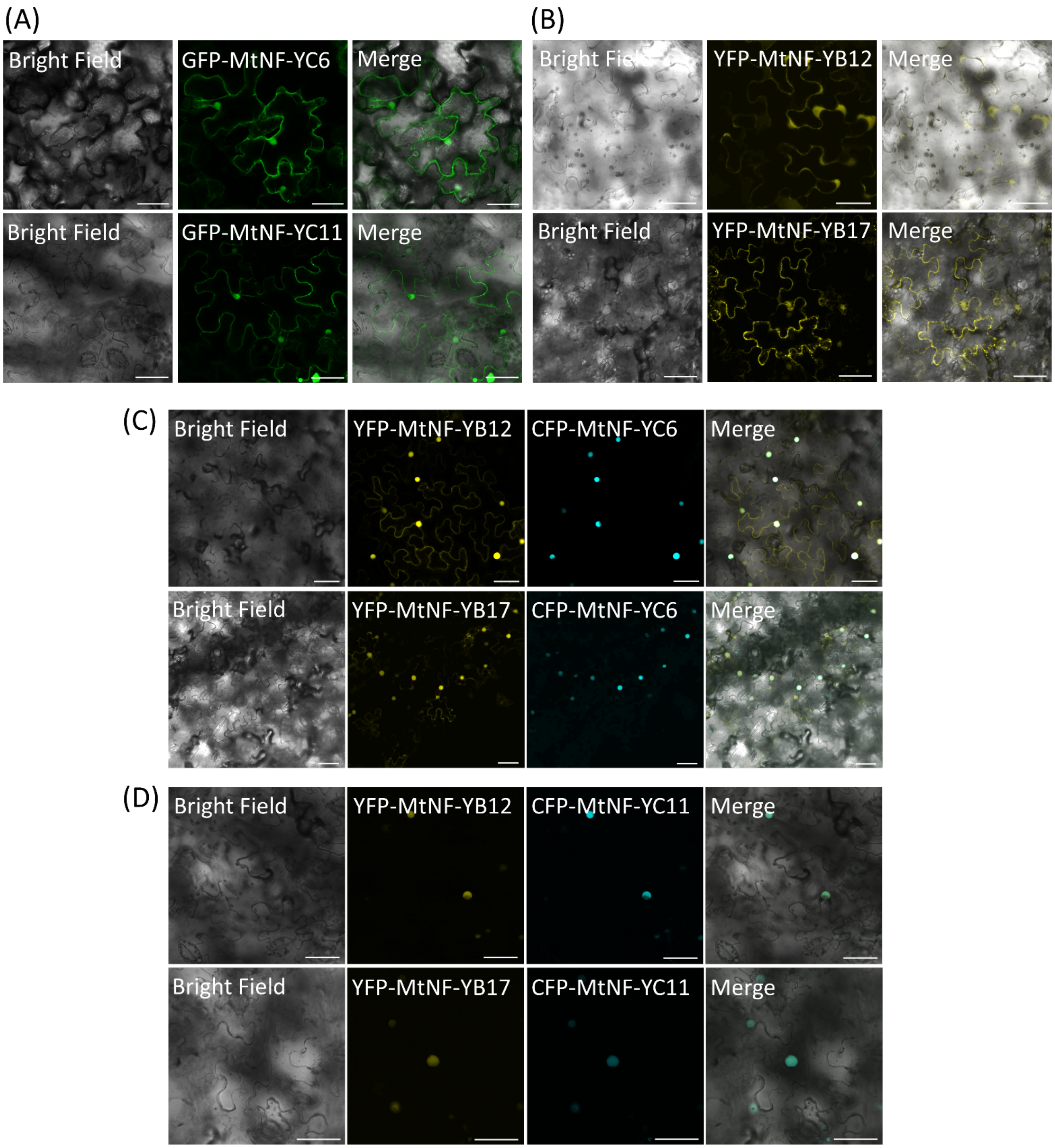

Supplementary Figure 3. Subcellular localization and colocalization of MtNF-YC6/C11 and MtNF-YB12/B17. (A) Subcellular localization of GFP-tagged MtNF-YC6 and MtNF-YC11. (B) Subcellular localization of YFP-tagged MtNF-YB12 and MtNF-YB17. (C) & (D) Co-expressing YFP-tagged MtNF-YB12 or MtNF-YB17 either with CFP-tagged MtNF-YC6 (C) or MtNF-YC11 (D). Merged images were the overlay of bright field and fluorescence signals. Bar = 50 µm.

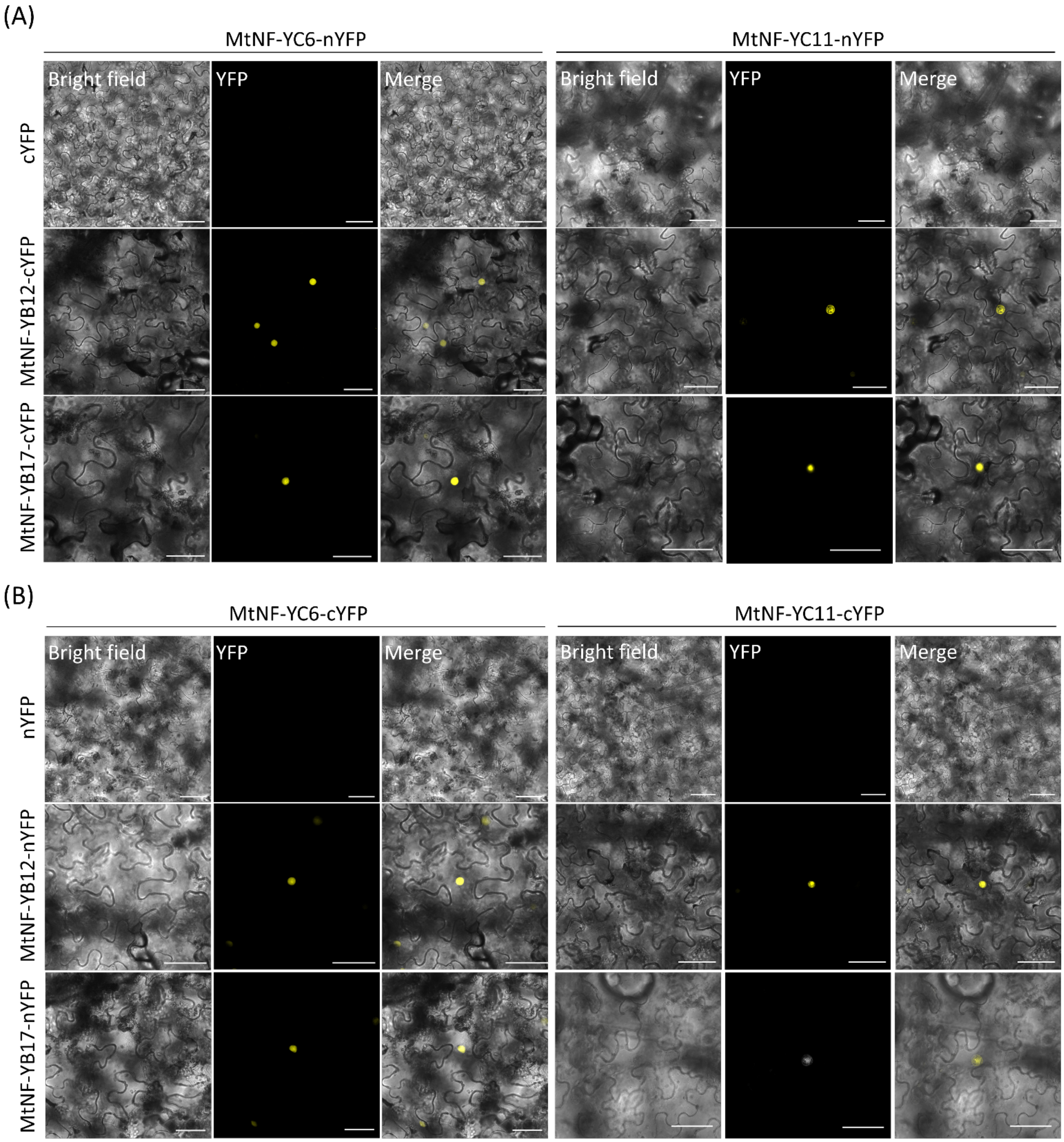

Supplementary Figure 4. The interaction between MtNF-YC6/C11 and MtNF-YB12/B17 was tested by BiFC assay. Merged images were the overlay of bright field and fluorescence signals. Bar = 50 μm.

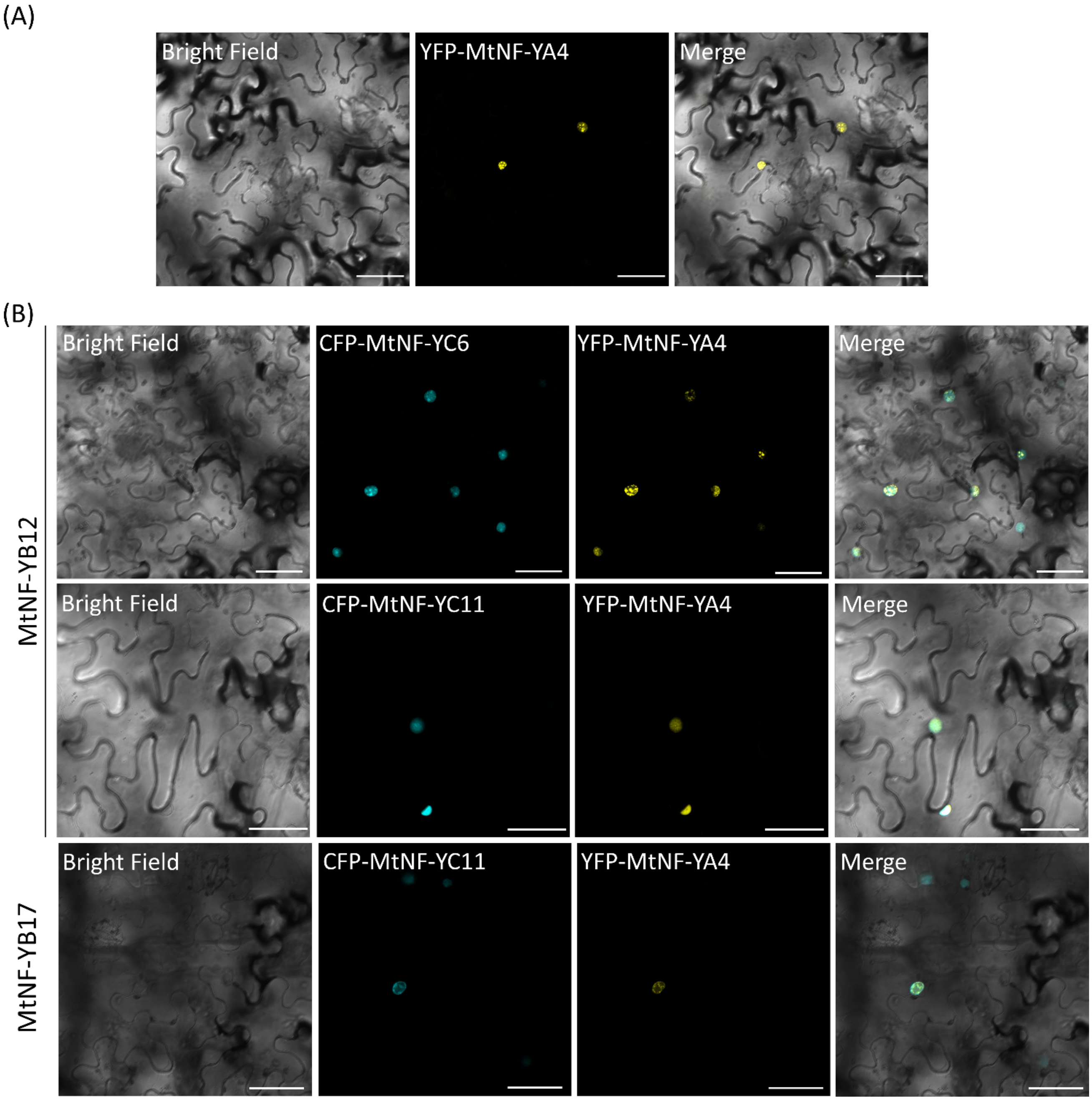

Supplementary Figure 5. The subcellular localization of YFP-MtNF-YA4 (A) and colocalization of YFP-MtNF-YA4 with CFP-MtNF-YC6 or CFP-MtNF-YC11 (B). Merged images were the overlay of bright field and fluorescence signals. Bar = 50 μm.

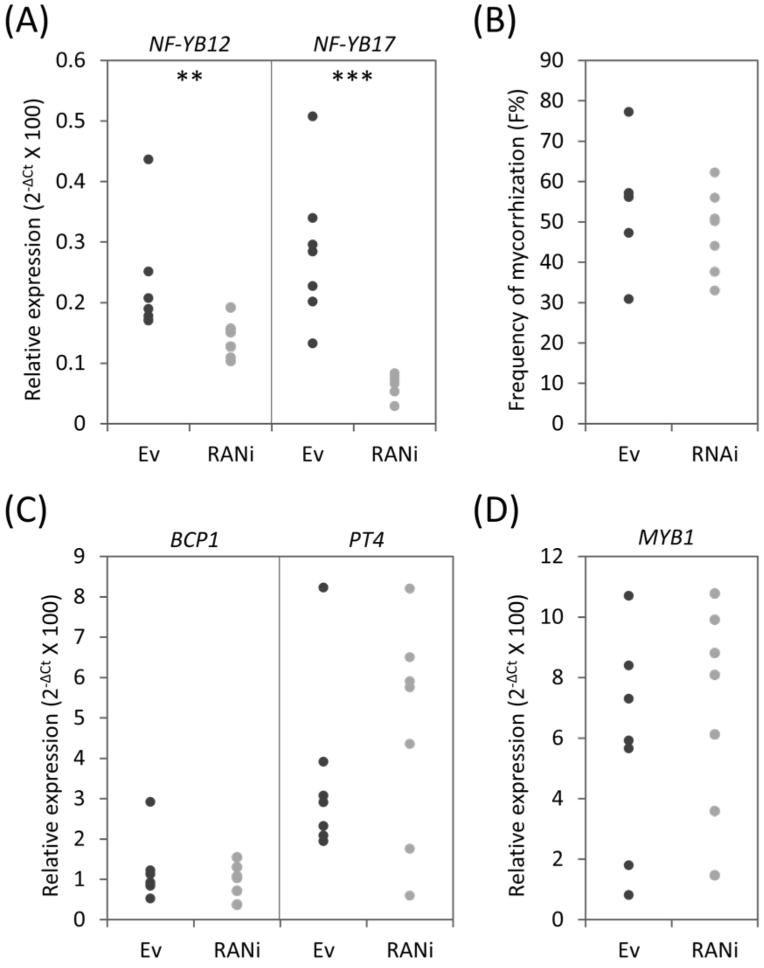

Supplementary Figure 6. The phenotypic analysis of *MtNF-YB17* RNAi roots. (A) The relative expression of *MtNF-YB12* and *MtNF-YB17* in empty vector control (Ev) and RNAi roots (RNAi). (B) The frequency of colonization in Ev and RNAi roots. (C) The relative expression of AMS marker genes. (D) The relative expression of *MtMYB1*. n = 7. Student's t test evaluated the difference between Ev and RNAi roots. **, $p<0.01$, ***, $p<0.001$.

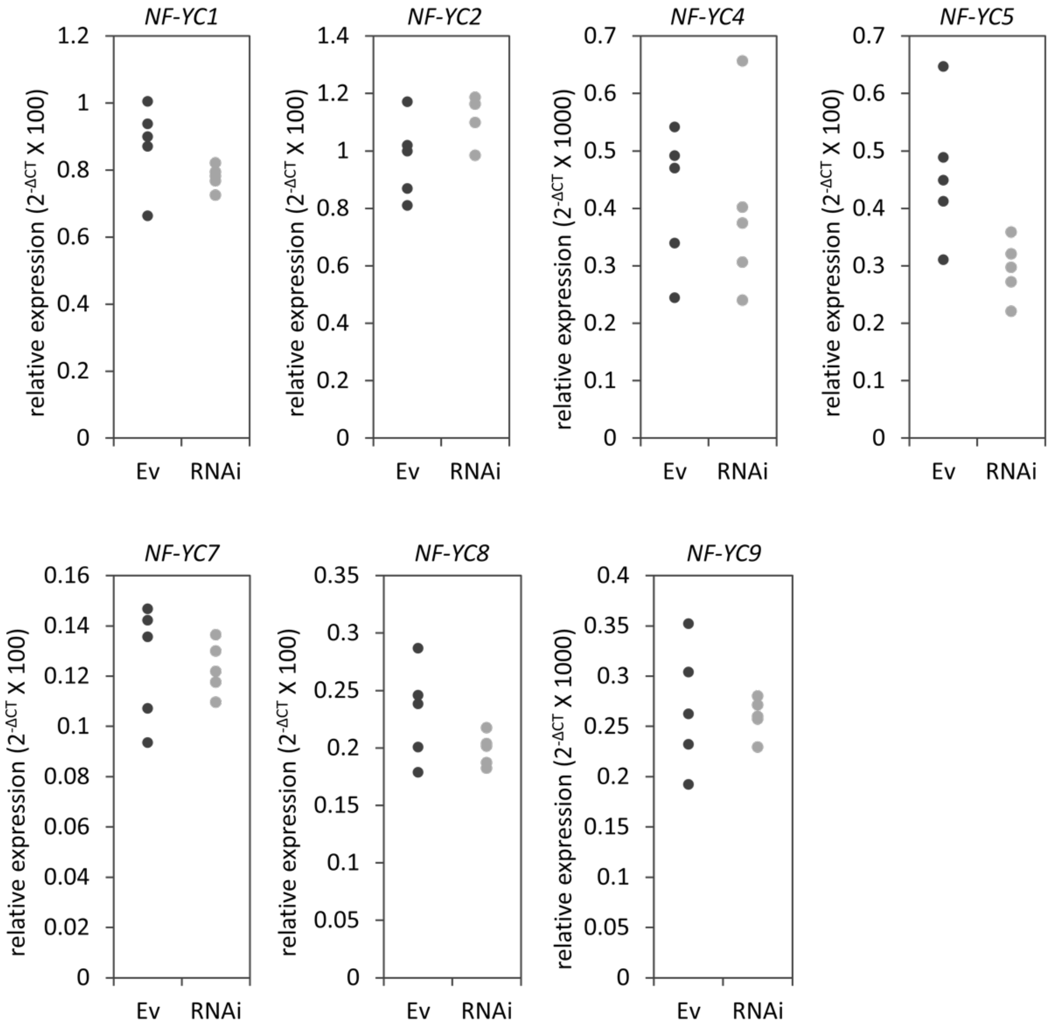

Supplementary Figure 7. The expression of *MtNF-YC* family members in empty vector control (Ev) and *MtNF-YC6/C11* RNAi roots (RNAi). n=5.

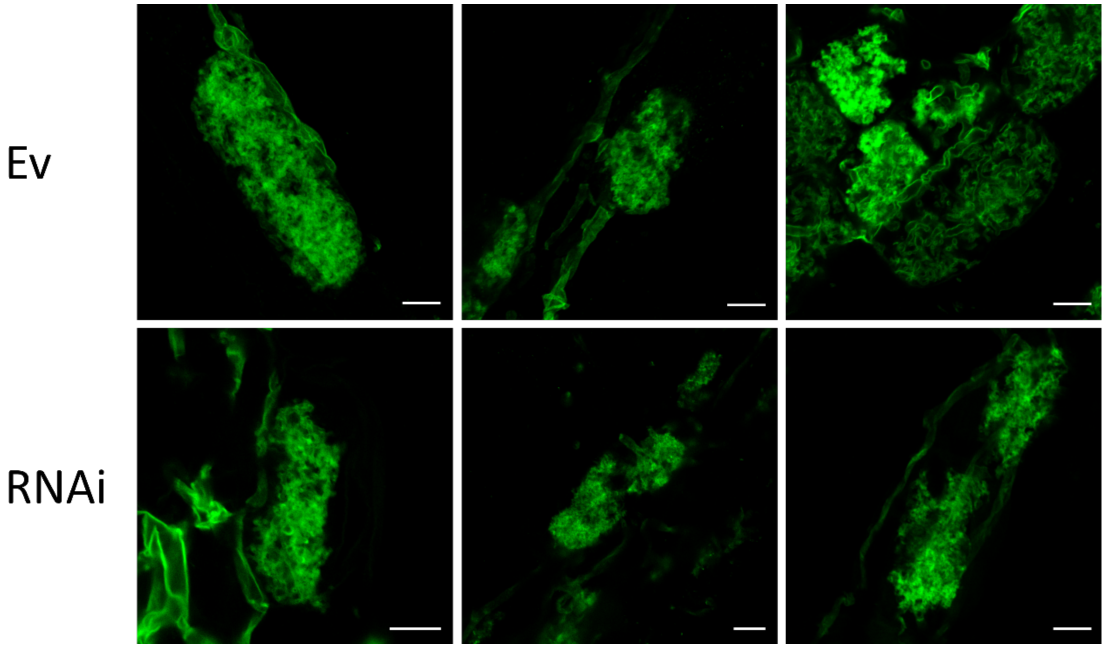

Supplementary Figure 8. The morphology of arbuscules in empty vector control and *MtNF-YC6/C11* RNAi roots. Bar = 10 µm.
